# Design and implementation of origami robot ROS-based SLAM and autonomous navigation

**DOI:** 10.1371/journal.pone.0298951

**Published:** 2024-03-28

**Authors:** Lijuan Zhao, Tianyi Zhang, Zuen Shang

**Affiliations:** 1 School of mechanical engineering, Liaoning Technical University, Fuxin, China; 2 The State Key Lab of Mining Machinery Engineering of Coal Industry, Liaoning Technical University, Fuxin, China; University of Liverpool, UNITED KINGDOM

## Abstract

In this study an innovative parameterized water-bomb wheel modeling method based on recursive solving are introduced, significantly reducing the modeling workload compared to traditional methods. A multi-link supporting structure is designed upon the foundation of the water-bomb wheel model. The effectiveness of the supporting structure is verified through simulations and experiments. For robots equipped with this water-bomb wheel featuring the multi-link support, base on the kinematic model of multi-link structure, a mapping algorithm that incorporates parameterized kinematic solutions and IMU-fused parameterized odometry is proposed. Based on this algorithm, SLAM and autonomous navigation experiments are carried out in simulation environment and real environment respectively. Compared with the traditional algorithm, this algorithm the precision of SLAM is enhanced, achieving high-precision SLAM and autonomous navigation with a robot error rate below 5%.

## Introduction

Due to the diversity of terrain structures and the unreasonable design of robot structures, existing rescue robots have difficulty adapting to post-disaster terrain and environments. Therefore, there are currently few successful cases of rescue robots performing rescue tasks. Mobile robots are mainly divided into wheeled, legged, tracked, and hybrid types [1, 2]. Compared with wheeled robots, legged-wheeled robots have better passability, and compared with legged robots, they have higher stability, combining all the advantages of wheeled and legged robots [3]. Therefore, legged-wheeled robots are considered to be one of the important directions for the development of rescue robots. However, current legged-wheeled rescue robots still face some challenges, such as difficulty entering narrow spaces and crossing obstacles. Therefore, innovative structural design for rescue robots is crucial.

Origami design provides new perspectives for the structural design of rescue robots.Many scholars in the field of origami design have achieved substantial theoretical results. Lu and colleagues successfully addressed the issue of planar configuration singularity in the folding process of origami patterns by introducing a non-linear predictive correction method and a spatial exploration algorithm for the four-fold paper [4]. Sareh explores the design of the least symmetric derivative of the Miura fold pattern, addressing challenges in flat-foldability for less symmetric descendants, and presents analytical solutions for the general problem of flat sheets with quadrilateral shapes on fold lines through their vertices [5]. Fonseca and colleagues, through the study of the dynamic characteristics of a self-folding robotic wheel driven by shape memory alloys, have revealed its complex nonlinear behaviors under various external stimuli, including chaos, transient chaos, and synchronization. This research establishes a theoretical foundation for enhancing the maneuverability and energy efficiency of autonomous robots [6]. Liu and colleagues, by studying a Miura-folded octagonal tube with bending, have provided an effective approach to enhance its energy absorption capacity by improving the folding scheme [7]. Hu and collaborators innovatively transformed origami models into fully programmable robotic systems using 3D printing technology. They introduced a folded spring model, providing a hierarchically clear programming approach for robot deformation adjustment [8]. These achievements in origami design have laid the theoretical foundation for the design of deformable structures.

In the specific applications of origami structures, rigid origami structures are widely used in robot design due to their excellent folding properties [9]. Professor Yan Chen, Professor Huijuan Feng, and others have conducted in-depth research on the rigid folding motion characteristics of water-bomb origami tubes [10–12], and summarized the folding robots. Professor Hongbin Fang has studied the earthworm-inspired robot, simulating the creeping motion of earthworms through the periodic axial and radial deformation of water-bomb origami structures [13]. Le et al. combined the art of origami with unmanned helicopters and compactly integrated the "origami suspension system" into the drone [14]. Shuguang Li’s team proposed a fluid-driven artificial muscle based on the folding structure, which can be used in wearable robotic exoskeletons, foldable space exploration structures, and many other fields [15]. Martinez et al. developed a driver consisting of a flexible composite material that uses the folding structure as an integral structure [16]. GE Healthcare collaborated with Brigham Young University to design an origami shield for the external extension arm of an X-ray machine used by doctors during surgery [17]. Miyashita et al. designed a miniature robot that can perform various clinical procedures inside the body under remote control by medical professionals [18]. The Matthew A. R. team developed a new modular robot platform, which realizes the driver of a "water-bomb" basic unit with three independent controls and embeds it in a parallel motion mechanism [19]. The Shuguang L. team developed a lightweight vacuum-driven soft robot gripper [20]. SHUO ZHANG and collaborators successfully employed a strategy involving laser scanning and injection of active particles to programmatically design features and functions on the surface of an elastic material. This innovative approach provides a means for manufacturing functional, soft ferromagnetic origami robots with seamless integrated structures and various active functionalities [21]. Yan and colleagues achieved the integration of autonomous folding robots by combining flexible bistable mechanisms and thermally conductive artificial muscles to create a folded multi-path switch. This setup allowed for the configuration of digital logic gates and storage units, showcasing diverse applications such as a flytrap-inspired fly-catching robot, an obstacle-avoiding crawler, and a wheeled vehicle [22]. The research in the application aspects of origami structures demonstrates their significant potential in the field of robotics.

As a special mechanism, the water-bomb origami variable-diameter wheel belongs to a type of walking wheel. Due to the segmented rim, it can generate a large traction force. Sa-Reum Kim and others designed a folding wheel robot called SNUMAX [23], which was made using a laminating process. Young Lee and others developed a dual-magic-ball-wheel vehicle [24] by driving a magic-ball wheel made of polyimide film and paper with a heated shape-memory alloy spiral spring and a passive spring. Professor Zhao Lijuan’s team analyzed and optimized the water-bomb structure wheel used in coal mine rescue robots [25]. However, as a thin-shell structure, the water-bomb structure still faces some challenges [26]. First, the modeling of the water-bomb structure requires multiple iterations [27], and the modeling process involves a large workload and needs to be optimized. In addition, the water-bomb structure is prone to problems such as cracking under heavy load conditions, and it needs to be finely designed and reinforced.

In addition, SLAM (Simultaneous Localization and Mapping) and path planning are crucial for the autonomous movement of rescue robots. Numerous scholars have made significant advancements in the fields of SLAM and path planning.Shen and colleagues compared three laser-based 2D SLAM algorithms (gapping, Hector-SLAM, and Cartographer) and discussed the strengths and weaknesses of each algorithm [28]. Zhang and collaborators, by comparing three SLAM algorithms and integrating a path planning analysis to assess their applicability in indoor rescue environments, have provided guidance for researchers in the construction of SLAM systems [29]. Lei improved the FastSLAM algorithm framework by introducing virtual particles as a global optimization objective and utilizing a particle swarm optimization approach [30]. Mu and colleagues proposed a graph-based multi-sensor SLAM (Simultaneous Localization and Mapping) method. They demonstrated the feasibility and effectiveness of this approach by integrating laser rangefinders, RGB-D cameras, encoders, and inertial measurement units through theoretical derivation and practical experiments [31]. Sombolestan and colleagues proposed a mobile robot path planning method based on integrated reinforcement learning. They compared it with commonly used methods, validating the effectiveness of this approach [32].Wang and colleagues introduced a dynamic path planning algorithm based on the Tree Double Deep Q Network to address the path planning problem for wheeled robots on sloped terrain with dynamic moving obstacles [33]. Xiang and colleagues proposed an enhanced Dynamic Window Approach (DWA) algorithm, incorporating a fuzzy controller to achieve adaptive weighting coefficients. This modification aims to make the robot’s path smoother during obstacle avoidance, enabling it to adapt to more complex environments [34]. Bo Zhang and colleagues proposed an algorithm that combines terrain feature modeling with the A* algorithm for global path planning. This algorithm has shown satisfactory results in avoiding local obstacles [35]. Borkar and colleagues introduced a path planning algorithm for wheeled robots in narrow streets using Generative Adversarial Networks. This algorithm utilizes deep learning methods to achieve efficient navigation [36]. However, current SLAM and path planning algorithms are primarily designed for traditional robots, and further in-depth research is needed for SLAM and path planning algorithms tailored to deformable-wheeled robots.

This article first proposes a water-bomb wheel modeling method to simplify the modeling process. Then, based on the water-bomb wheel model, a variable diameter wheel combining multiple linkages and water-bomb structures is designed to increase the strength of the water-bomb wheel. A variable diameter wheel robot is designed based on this special variable diameter wheel. Due to its variable diameter characteristics, the kinematic model of the robot varies greatly in different diameter states. Existing fixed kinematic solving methods cannot solve the kinematic problems of the robot in various states. At the same time, the robot’s autonomous navigation system relies on odometer information and kinematic solving methods. This article proposes mapping algorithm that incorporates parameterized kinematic solutions and IMU-fused parameterized odometry to achieve SLAM and autonomous navigation in ROS-based simulation environments and physical prototypes.

## Modeling of water-bomb wheels

**Fig 1:**
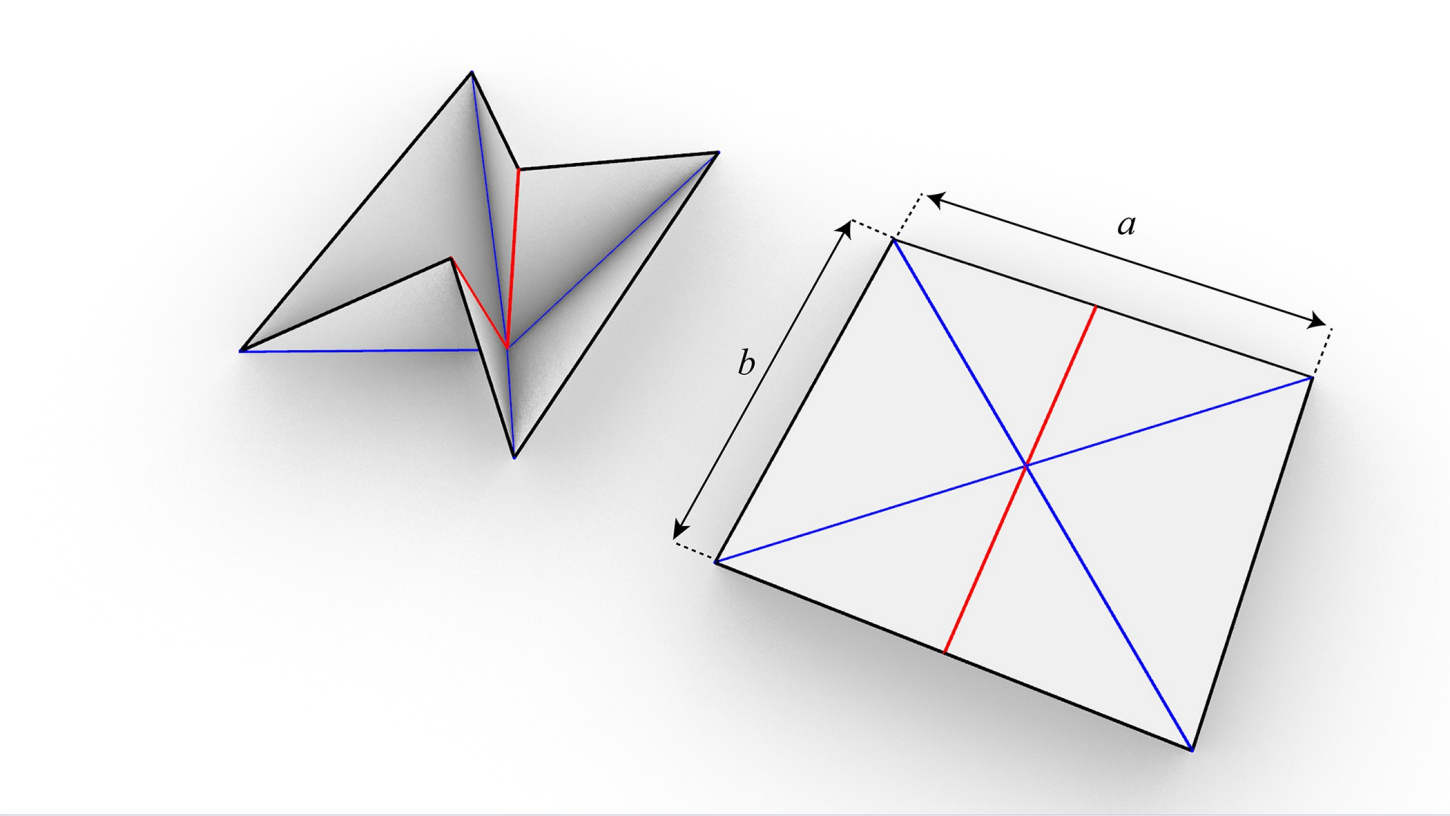
Basic unit of water-bomb.

**Fig 2:**
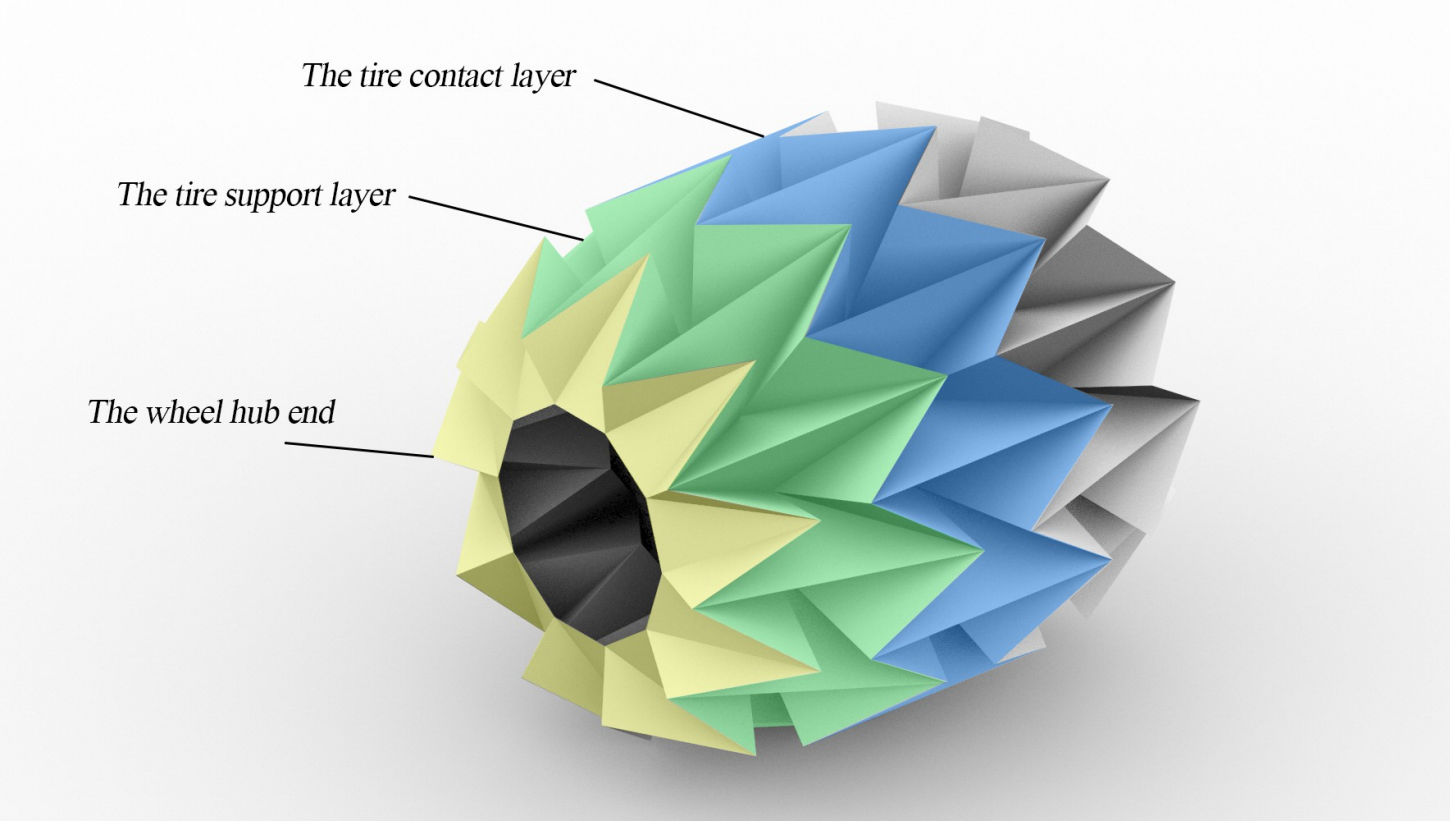
Appearance drawing of water-bomb wheel.

The modeling of water bomb wheel is a complex and important work.Taking a water-bomb wheel composed of three layers of basic units as an example, Fig 1 shows the basic unit of the water-bomb fold, a and b which represents the width and height of the water-bomb unit, respectively. The water-bomb wheel can be divided into the wheel axle end layer, wheel support layer, and wheel contact layer as a whole, as shown in Fig 2 for the appearance of the water-bomb wheel.

Traditional water-bomb wheel modeling methods often require solving a system of over ten equations globally to determine the coordinates of all points at once. Generic solvers struggle to handle such problems, and this system of equations can have multiple solutions. On one hand, due to the limited precision of global optimization numerical methods, the model accuracy is wired.On the other hand the traditional process of solving these huge equations using numerical methods, it is hard to filter out the specific solutions that meet our unique requirements. These makes traditional modeling methods highly challenging.

To address this challenge, this paper introduces a novel recursive-based modeling approach. This method, based on the coordinates of three known points, calculates the coordinates of an unknown point, reducing the number of equations in a single solve to three. These equations can be solved using traditional solvers or even analytically, enhancing modeling accuracy and reducing the workload in the modeling process.

**Fig 3:**
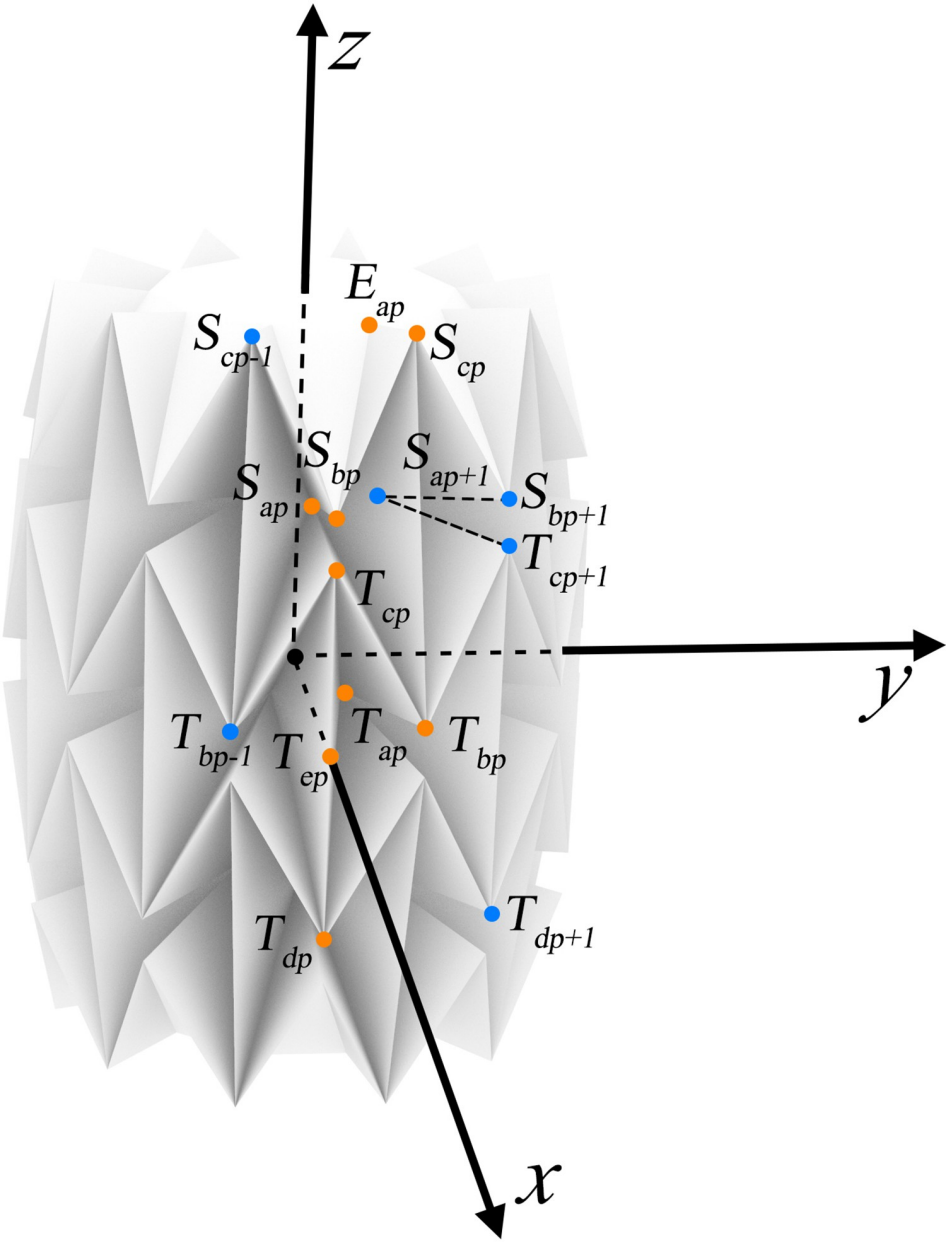
Rectangular coordinate system of the water-bomb.

**Fig 4:**
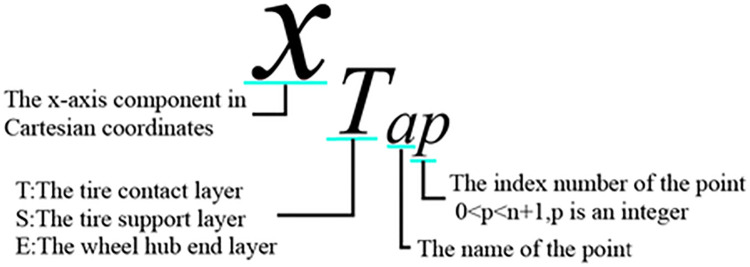
Explanation of letters and subscripts.

The axis of the water-bomb wheel is considered as the z-axis, and the x-axis passes through the midpoint of the line connecting *T*_*cp*_ and *T*_*dp*_(*T*_*ep*_), as shown in Fig 3. *α*_*1*_ represents the inclination angle of the units in the wheel contact layer, while *α*_*2*_ represents the inclination angle of units in the wheel support layer, a and b represents the units width and height. The modeling method described in this article uses the dihedral angle *θ* between the surface *T*_*ap*_, *T*_*cp*_, *T*_*dp*_, and the surface *T*_*ap*_, *T*_*cp+1*_, *T*_*dp+1*_ as the state parameter. Fig 4 provides an explanation of the symbols used, where x represents the x-coordinate value, T represents the wheel support layer, a represents the specific name of the point, and p represents the point number (0<p<n+1).

Analyzing the motion patterns of the wheel contact layer, it is not difficult to obtain the 3D coordinates of points *T*_*ep*_ and *T*_*ap*_. Based on the 3D coordinates of points *T*_*ep*_ and *T*_*ap*_, along with shape parameters n, *a*, *b*, *θ*, *α*_*1*_, and *α*_*2*_, we can recursively calculate the 3D coordinates of every point on the water-bomb wheel. It is not difficult to determine the 3D coordinates of *T*_*ep*_ and *T*_*ap*_:

$$T_{ep}:\;[\begin{array}{l} x_{T_{cp}}\\ y_{T_{\mathrm{cp}}}\\ z_{T_{\mathrm{cp}}} \end{array}]=[\begin{array}{c} \cos(\frac{2p\pi }{n})\cdot \frac{a}{2}\cdot \sin(\frac{\theta }{2})/\sin(\frac{\pi }{n})\\ \sin(\frac{2p\pi }{n})\cdot \frac{a}{2}\cdot \sin(\frac{\theta }{2})/\sin(\frac{\pi }{n})\\ 0 \end{array}]$$

$$T_{ap}:\;[\begin{array}{l} x_{T_{\mathrm{ap}}}\\ y_{T_{ap}}\\ z_{T_{ap}} \end{array}]=[\begin{array}{c} \cos(\frac{2p\pi }{n}+\frac{\pi }{n})\cdot [\frac{a}{2}\cdot \sin(\frac{\theta }{2})/\sin(\frac{\pi }{n})\cdot \cos(\frac{\pi }{n})-\frac{a}{2}\cos(\frac{\theta }{2})]\\ \sin(\frac{2p\pi }{n}+\frac{\pi }{n})\cdot [\frac{a}{2}\cdot \sin(\frac{\theta }{2})/\sin(\frac{\pi }{n})\cdot \cos(\frac{\pi }{n})-\frac{a}{2}\cos(\frac{\theta }{2})]\\ 0 \end{array}]$$

The point *T*_*cp*_ can be derived from *T*_*ep*_, *T*_*ap*_ and *T*_*ap+1*_ by solving the following equations:

$${\left(x_{T_{cp}}-x_{T_{ep}}\right)}^{2}+{\left(y_{T_{cp}}-y_{T_{ep}}\right)}^{2}+{\left(z_{T_{cp}}-z_{T_{ep}}\right)}^{2}-\frac{b^{2}}{4}=0$$

$${\left(x_{T_{cp}}-x_{T_{\mathrm{ap}}}\right)}^{2}+{\left(y_{T_{cp}}-y_{T_{ap}}\right)}^{2}+{\left(z_{T_{cp}}-z_{T_{ap}}\right)}^{2}-\frac{{\left(a-b*\cos(pi/2-\alpha_{1})\right)}^{2}+{\left(b*\sin(pi/2-\alpha_{1})\right)}^{2}}{4}=0$$

$${\left(x_{T_{cp}}-x_{T_{ap-1}}\right)}^{2}+{\left(y_{T_{cp}}-y_{T_{ap-1}}\right)}^{2}+{\left(z_{T_{cp}}-z_{ap-1}\right)}^{2}-\frac{{\left(a+b*\cos(pi/2-\alpha_{1})\right)}^{2}+{\left(b*\sin(pi/2-\alpha_{1})\right)}^{2}}{4}=0$$

The point *T*_*bp*_ can be derived from *T*_*ap*_, *T*_*cp*_ and *T*_*cp+1*_ by solving the following g equations:

$$\begin{array}{c} {\left(x_{T_{bp}}-x_{T_{ap}}\right)}^{2}+{\left(y_{T_{bp}}-y_{T_{ap}}\right)}^{2}+{\left(z_{T_{bp}}-z_{T_{ap}}\right)}^{2}-\frac{b^{2}}{4}=0\\ {\left(x_{T_{bp}}-x_{T_{cp}}\right)}^{2}+{\left(y_{T_{bp}}-y_{T_{cp}}\right)}^{2}+{\left(z_{T_{bp}}-z_{T_{cp}}\right)}^{2}-\frac{a^{2}}{4}=0\\ {\left(x_{T_{bp}}-x_{T_{cp+1}}\right)}^{2}+{\left(y_{T_{bp}}-y_{T_{cp+1}}\right)}^{2}+{\left(z_{T_{bp}}-z_{cp+1}\right)}^{2}-\frac{a^{2}}{4}=0 \end{array}$$

The point *S*_*ap*_ can be derived from *T*_*bp*_, *T*_*bp-1*_ and *T*_*cp*_ by solving the following equations:

$${\left(x_{S_{ap}}-x_{T_{bp}}\right)}^{2}+{\left(y_{S_{ap}}-y_{T_{bp}}\right)}^{2}+{\left(z_{S_{ap}}-z_{T_{bp}}\right)}^{2}-\frac{{\left(a-b*\cos(pi/2-\alpha_{2})\right)}^{2}+{\left(b*\sin(pi/2-\alpha_{2})\right)}^{2}}{4}=0$$

$${\left(x_{S_{ap}}-x_{T_{bp-1}}\right)}^{2}+{\left(y_{S_{ap}}-y_{T_{bp-1}}\right)}^{2}+{\left(z_{S_{ap}}-z_{T_{bp-1}}\right)}^{2}-\frac{{\left(a+b*\cos(pi/2-\alpha_{2})\right)}^{2}+{\left(b*\sin(pi/2-\alpha_{2})\right)}^{2}}{4}=0$$

$${\left(x_{S_{ap}}-x_{T_{cp}}\right)}^{2}+{\left(y_{S_{ap}}-y_{T_{cp}}\right)}^{2}+{\left(z_{S_{ap}}-z_{T_{cp}}\right)}^{2}-\frac{b^{2}}{4}=0$$

The point *S*_*cp*_ can be derived from *T*_*ap*_, *T*_*ap+1*_ and *T*_*bp*_ by solving the following equations:

$${\left(x_{S_{cp}}-x_{T_{bp}}\right)}^{2}+{\left(y_{S_{cp}}-y_{T_{bp}}\right)}^{2}+{\left(z_{S_{cp}}-z_{T_{bp}}\right)}^{2}-b^{2}=0$$

$${\left(x_{S_{cp}}-x_{T_{ap+1}}\right)}^{2}+{\left(y_{S_{cp}}-y_{T_{ap+1}}\right)}^{2}+{\left(z_{S_{cp}}-z_{T_{ap+1}}\right)}^{2}-\frac{{\left(a-b*\cos(pi/2-\alpha_{2})\right)}^{2}+{\left(b*\sin(pi/2-\alpha_{2})\right)}^{2}}{4}=0$$

$${\left(x_{S_{cp}}-x_{T_{ap}}\right)}^{2}+{\left(y_{S_{cp}}-y_{T_{ap}}\right)}^{2}+{\left(z_{S_{cp}}-z_{T_{ap}}\right)}^{2}-\frac{{\left(a+b*\cos(pi/2-\alpha_{2})\right)}^{2}+{\left(b*\sin(pi/2-\alpha_{2})\right)}^{2}}{4}=0$$

The point *S*_*bp*_ can be derived from *S*_*cp*_, *S*_*cp-1*_ and *S*_*ap*_ by solving the following equations:

$$\begin{array}{c} {\left(x_{S_{bp}}-x_{S_{cp}}\right)}^{2}+{\left(y_{S_{bp}}-y_{S_{cp}}\right)}^{2}+{\left(z_{S_{bp}}-z_{S_{cp}}\right)}^{2}-\frac{a^{2}}{4}=0\\ {\left(x_{S_{bp}}-x_{S_{cp-1}}\right)}^{2}+{\left(y_{S_{bp}}-x_{S_{cp-1}}\right)}^{2}+{\left(z_{S_{bp}}-x_{S_{cp-1}}\right)}^{2}-\frac{a^{2}}{4}=0\\ {\left(x_{S_{bp}}-x_{S_{ap}}\right)}^{2}+{\left(y_{S_{bp}}-y_{S_{ap}}\right)}^{2}+{\left(z_{S_{bp}}-z_{S_{ap}}\right)}^{2}-\frac{b^{2}}{4}=0 \end{array}$$

The point *E*_*ap*_ can be derived from *S*_*bp*_, *S*_*bp+1*_ and *S*_*cp*_ by solving the following equations:

$$\begin{array}{c} {\left(x_{E_{ap}}-x_{S_{cp}}\right)}^{2}+{\left(y_{E_{ap}}-y_{S_{cp}}\right)}^{2}+{\left(z_{E_{ap}}-z_{S_{cp}}\right)}^{2}-\frac{b^{2}}{16}=0\\ {\left(x_{E_{ap}}-x_{S_{bp+1}}\right)}^{2}+{\left(y_{E_{ap}}-x_{S_{bp+1}}\right)}^{2}+{\left(z_{E_{ap}}-x_{S_{bp+1}}\right)}^{2}-\frac{4a^{2}+b^{2}}{16}=0\\ {\left(x_{E_{ap}}-x_{S_{bp}}\right)}^{2}+{\left(y_{E_{ap}}-y_{S_{bp}}\right)}^{2}+{\left(z_{E_{ap}}-z_{S_{bp}}\right)}^{2}-\frac{4a^{2}+b^{2}}{16}=0 \end{array}$$

**Fig 5:**
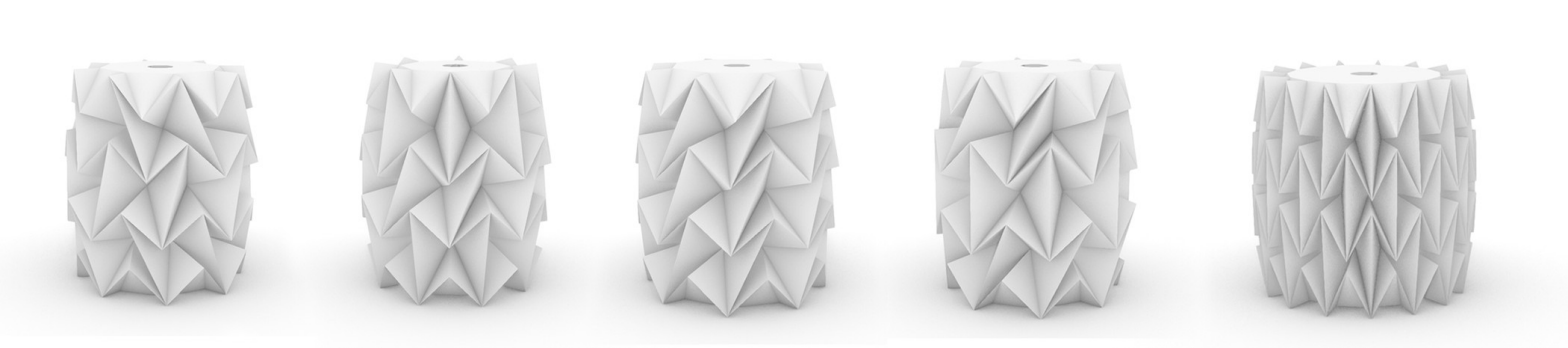
Different types of water-bomb wheels.

By using the equations mentioned above, we can solve for the coordinates of all points on the upper half of the water-bomb wheel, and similarly, we can determine the coordinates for the lower half of the water-bomb wheel. Utilizing these equations and varying shape and state parameters, we can easily perform parametric modeling of the water-bomb wheel. Fig 5 illustrates various types of water-bomb wheel models.

## Structural design of deformation wheel and kinematics analysis of robot

### Design of robot deformation wheel

**Fig 6:**
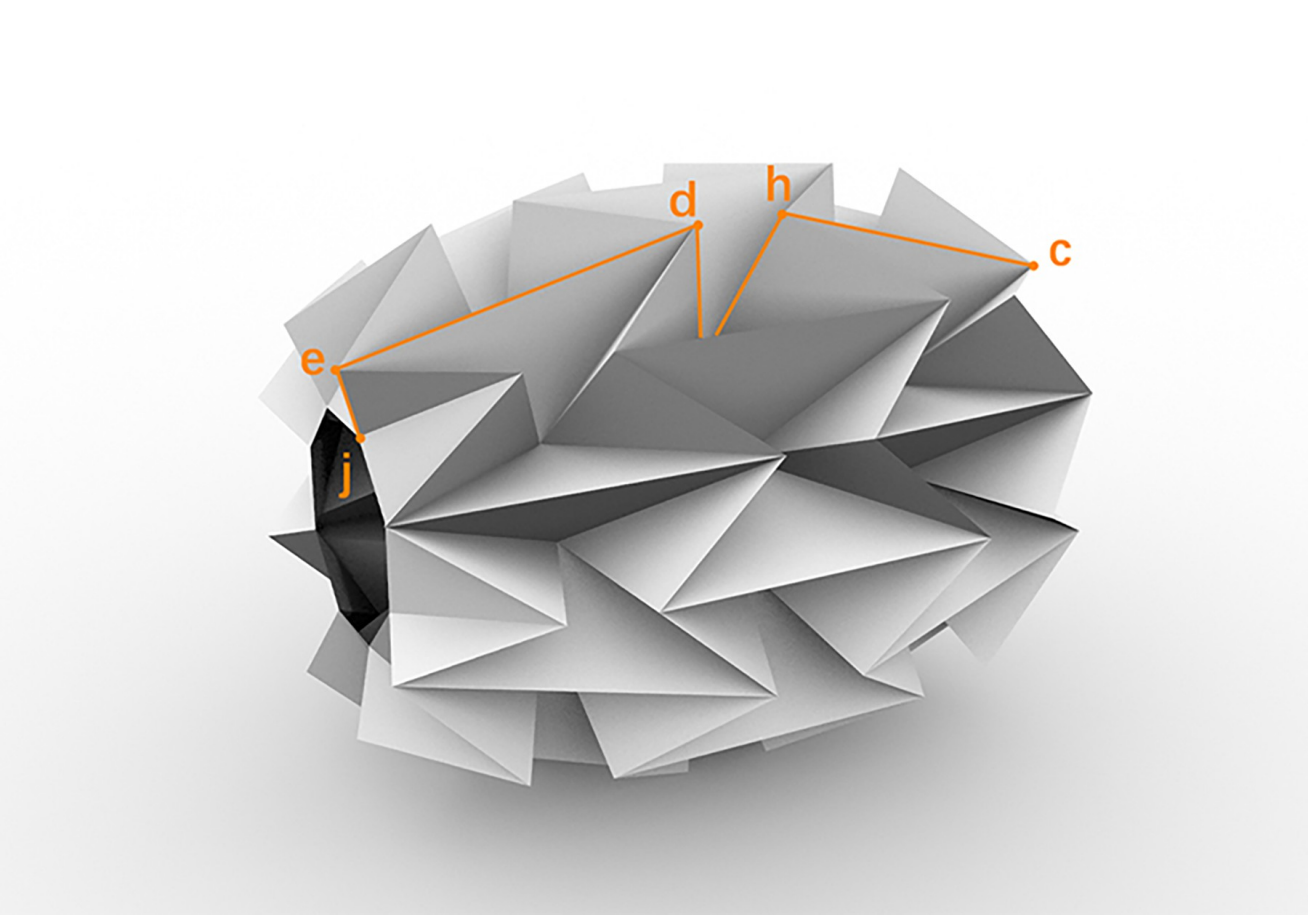
Schematic diagram of water-bomb wheel shell crease.

According to the kinematic model of the water-bomb wheel shown in Fig 6, point *d*, *h* is the main force point when the wheel rolls. In this paper, a water-bomb wheel consisting of 3x8 basic units is used. When the end face of the wheel is pushed in, the radius of the wheel increases. Due to the nature of the structure’s own motion, it is possible to control the shape of the wheel using only one actuator acting on the end face.

**Fig 7:**
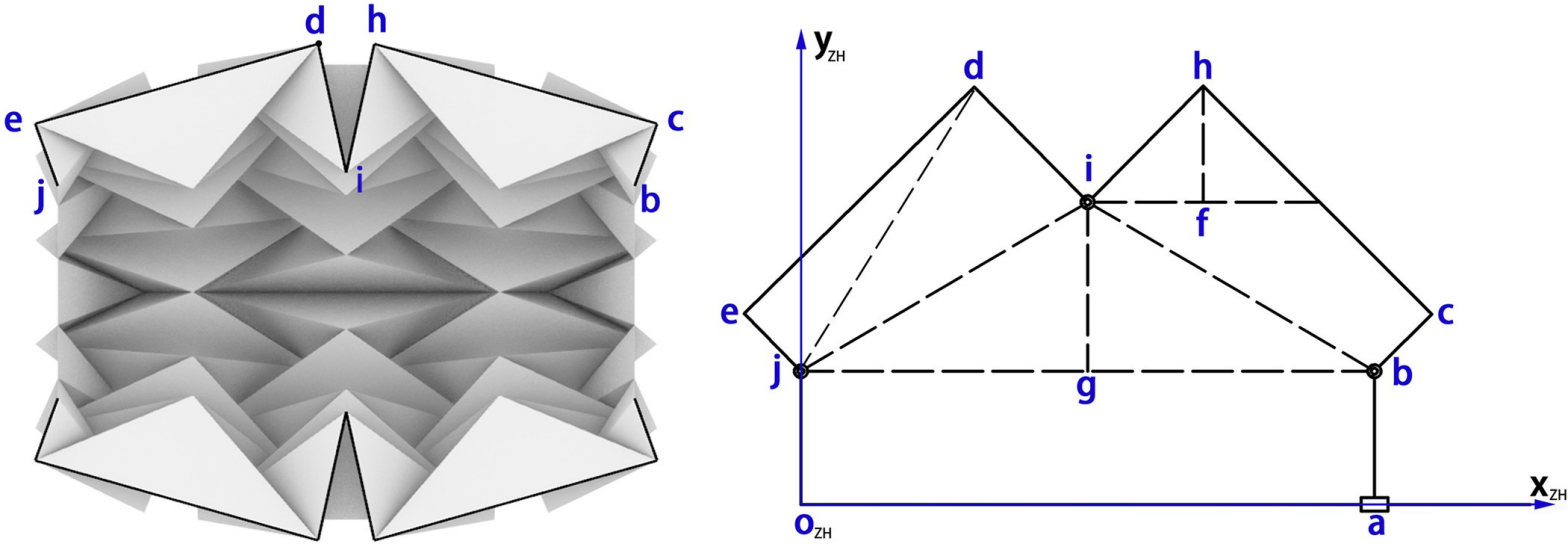
Kinematic analysis of mountain fold.

The mountain fold of the water-bomb wheel can be represented as a linkage and joint model. Fig 7 shows the kinematic model of the mountain fold in the *x*_*ZH*_*-y*_*ZH*_ plane. *l* represents half the length of the wheel, *s* represents the length of the basic component, and *r* represents the radius of the wheel spoke. Assuming an ideal rotational joint at point *i* of the mountain fold, due to the small and negligible changes in the angles *e*, *d*, *h*, and *c* around 90 degrees during the unfolding process, they can be ignored. The mountain fold can be modeled as two "C"-shaped rigid links, as shown in the simplified diagram in Fig 7. By analyzing the structure of the wheel in the kinematic model, the range of the wheel diameter variation can be obtained, and the required displacement of the driver can be determined. In the Fig 7, point *a* represents a slider that can slide freely on the extended center axis *oa*. *L*_*jd*_, *L*_*if*_, *L*_*ig*_, and *L*_*hf*_ represent the lengths of the lines *jd*, *if*, *ig*, and *hf*, respectively. *L*_*bc*_ *= L*_*ej*_ *= s*, *L*_*ed*_ *= L*_*hc*_ *= 4s*, *L*_*di*_ *= L*_*hi*_ *= 2s*, *∠hib = β*,*∠dji = γ*, *∠ijg = ∠ibj = α*. *x*_*d*_ and *y*_*d*_ represent the x-coordinate and y-coordinate of point d, respectively, and *x*_*h*_ and *y*_*h*_ represent the x-coordinate and y-coordinate of point *h*, respectively. The coordinates of point *d* and point *h* are as follows:

$$d:\;[\begin{array}{l} x_{d}\\ y_{d} \end{array}]=[\begin{array}{c} L_{jd}\cdot \cos(\gamma +\alpha )\\ L_{jd}\cdot \sin(\gamma +\alpha ) \end{array}]$$

$$h:\;[\begin{array}{l} x_{h}\\ y_{h} \end{array}]=[\begin{array}{c} 2s\cdot \cos(\beta -\alpha )+l\\ \sqrt{17s^{2}-l^{2}}+2s\cdot \sin(\beta -\alpha )+r \end{array}]$$

**Fig 8:**
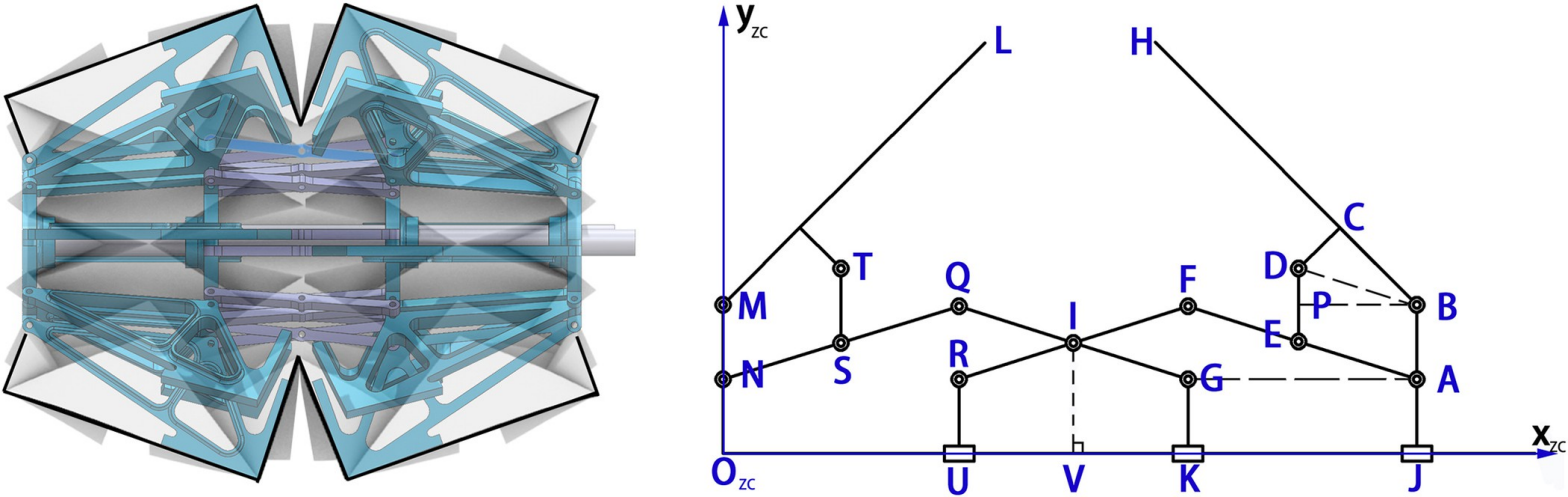
Kinematic analysis of support structure.

To improve the strength of the water-bomb wheel, a multilink bracket was designed to support its main force points, *d* and *h*, as shown in Fig 8. This structure aims to support the *d* and *h* ground contact points of the mountain folds along the *L* and *H* points of the magic ball wheel axis. Kinematic analysis was performed on the support structure’s *L* and *H* points: The sliders *U*, *K*, and *J* can slide along the central axis, and due to the structural characteristics, the "T"-shaped rod *MTL* can move by mirror symmetry about the *IV* line with the same size as the *BDH*. Here, *L*_*HB*_ = *L*_*ML*_ = *L*_*jd*_, *L*_*NQ*_ = *L*_*QG*_ = *L*_*RF*_ = *L*_*FA*_ = 2/3*L*_*ij*_, and the points *S*, *I*, and *E* are located at the center of rods *NQ*, *QG*, *RF*, and *FA*, respectively. Additionally, *L*_*DB*_ = *L*_*EA*_, *L*_*HB*_ = *L*_*jd*_, *L*_*MO*_ = *L*_*BJ*_ = *r*, *L*_*BA*_ = *L*_*MN*_ = *L*_*DE*_ = *L*_*TS*_ = 0.5*r*, *L*_*OV*_ = *L*_*VJ*_ = *l*, *∠DBH* = *γ*_*1*_, and *∠FAG = α*_*1*_. The coordinates of the L and H points are as follows:

$$H:\;[\begin{array}{l} x_{H}\\ y_{H} \end{array}]=[\begin{array}{c} 2l-\cos(\alpha_{1}+\gamma_{1})\cdot L_{HB}\\ r+\sin(\alpha_{1}+\gamma_{1})\cdot L_{HB} \end{array}]$$

$$L:\;[\begin{array}{l} x_{L}\\ y_{L} \end{array}]=[\begin{array}{c} \cos(\alpha +\gamma )\cdot L_{HB}\\ r+\sin(\alpha +\gamma )\cdot L_{HB} \end{array}]$$

Under the assumption of a double-C linkage, the water-bomb wheel shell can perfectly fit a multi-link support structure. However, in the actual folding process, the shape of the water-bomb wheel mountain fold is not always that of a perfect double-C linkage. To verify the specific fit between the multi-linkage and the water-bomb wheel shell, we set the parameter s = 37.5mm and used the modeling method proposed in this chapter to create a 3D model. We conducted folding experiments in Abaqus software and used Python scripts to extract end-to-end distances and diameter data during the folding process of the water-bomb wheel shell, recording the data.

**Fig 9:**
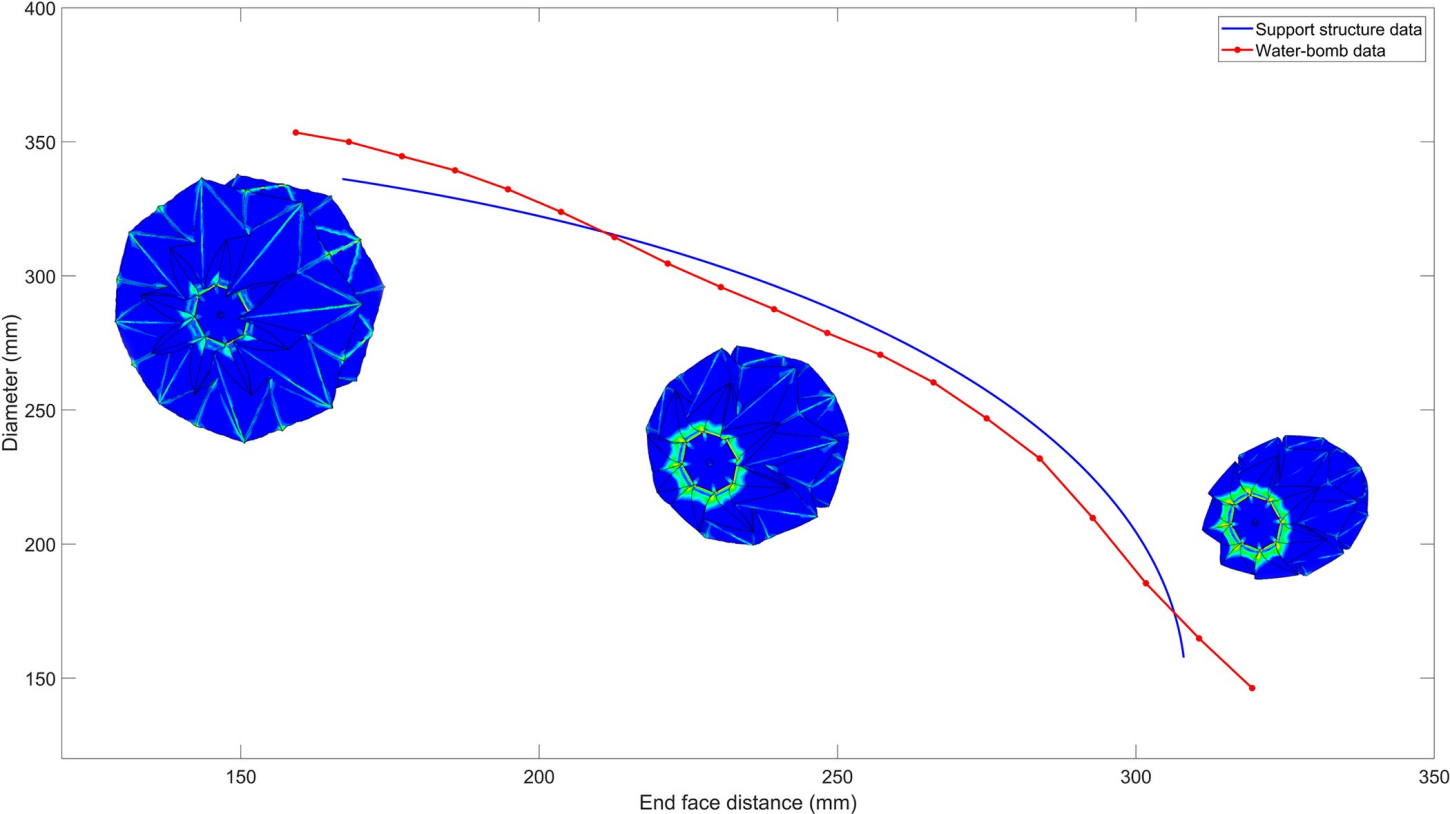
Comparison of simulation data between multi-link and water-bomb wheel.

Simultaneously, we used a mathematical model to calculate end-to-end distances and diameter data during the folding process of the multi-linkage structure and recorded that data as well. Comparing the two sets of data, as shown in Fig 9, when the end-to-end distances are the same, the maximum difference in diameter does not exceed 16mm. The simulation results indicate that a rigid support structure can effectively support the flexible water-bomb wheel shell.

**Fig 10:**
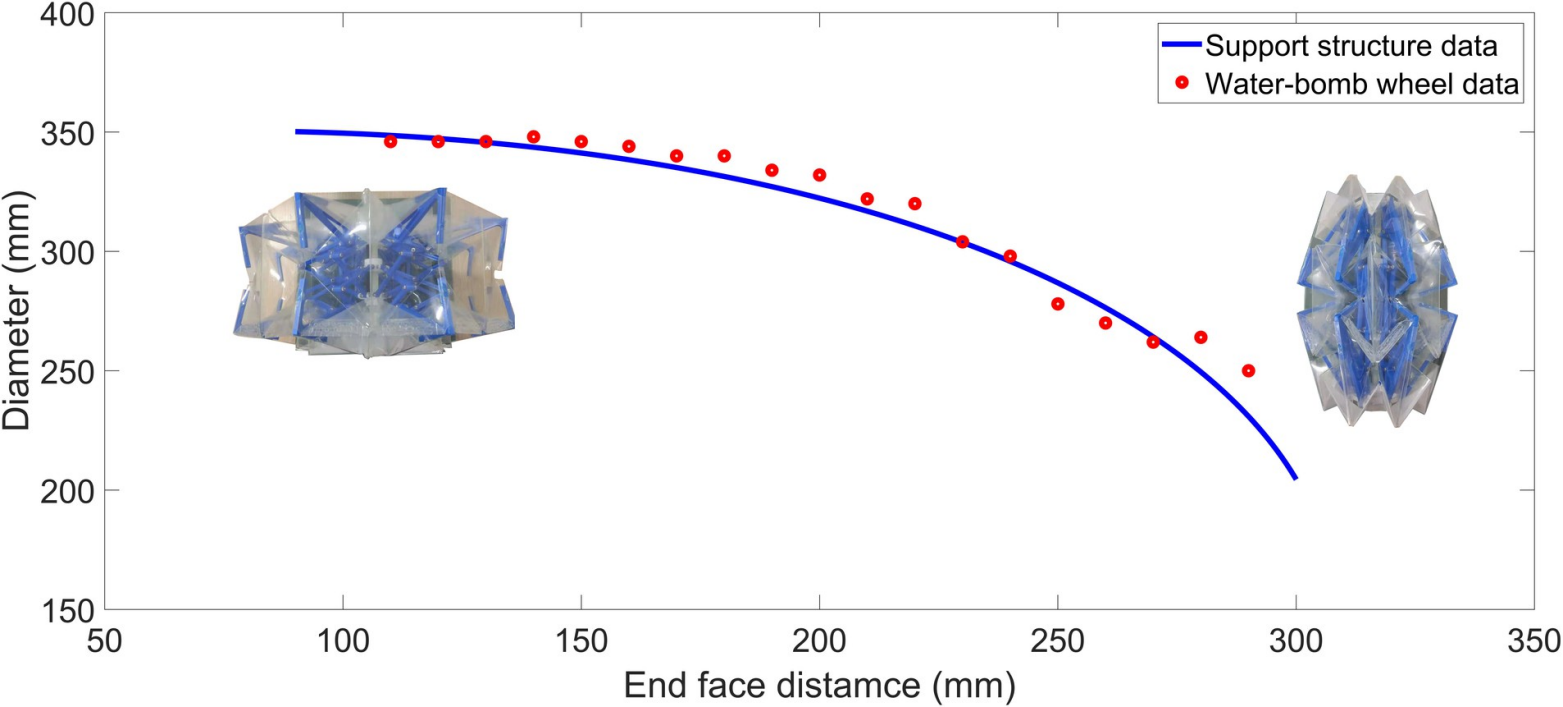
Comparison of actual data between multi-link and water-bomb wheel.

We used laser engraving on PET film to create fold lines for making the water-bomb wheel shell. Additionally, in the aforementioned ABAQUS simulation results, we observed that during the folding process, the wheel axle layer and wheel contact layer of the water-bomb wheel shell experienced significant stress at their vertices. Therefore, we used a heat press apparatus to reinforce the wheel axle layer of the water-bomb wheel shell with heat transfer adhesive and nylon mesh. In this area, stress-relief holes were also cut at the vertices of the wheel contact layer to reduce stress. Simultaneously, we utilized 3D printing technology to create a multi-link support structure. Combining these components to assemble the water-bomb wheel, we conducted experiments and measured the distance data between the end face and diameter during the folding process for 19 sets. This data was then compared to the mathematical model data for the support structure. As shown in Fig 10, the measurement results indicate that the support structure can effectively provide support at the target support points.

### Parametric kinematics solution of robot

**Fig 11:**
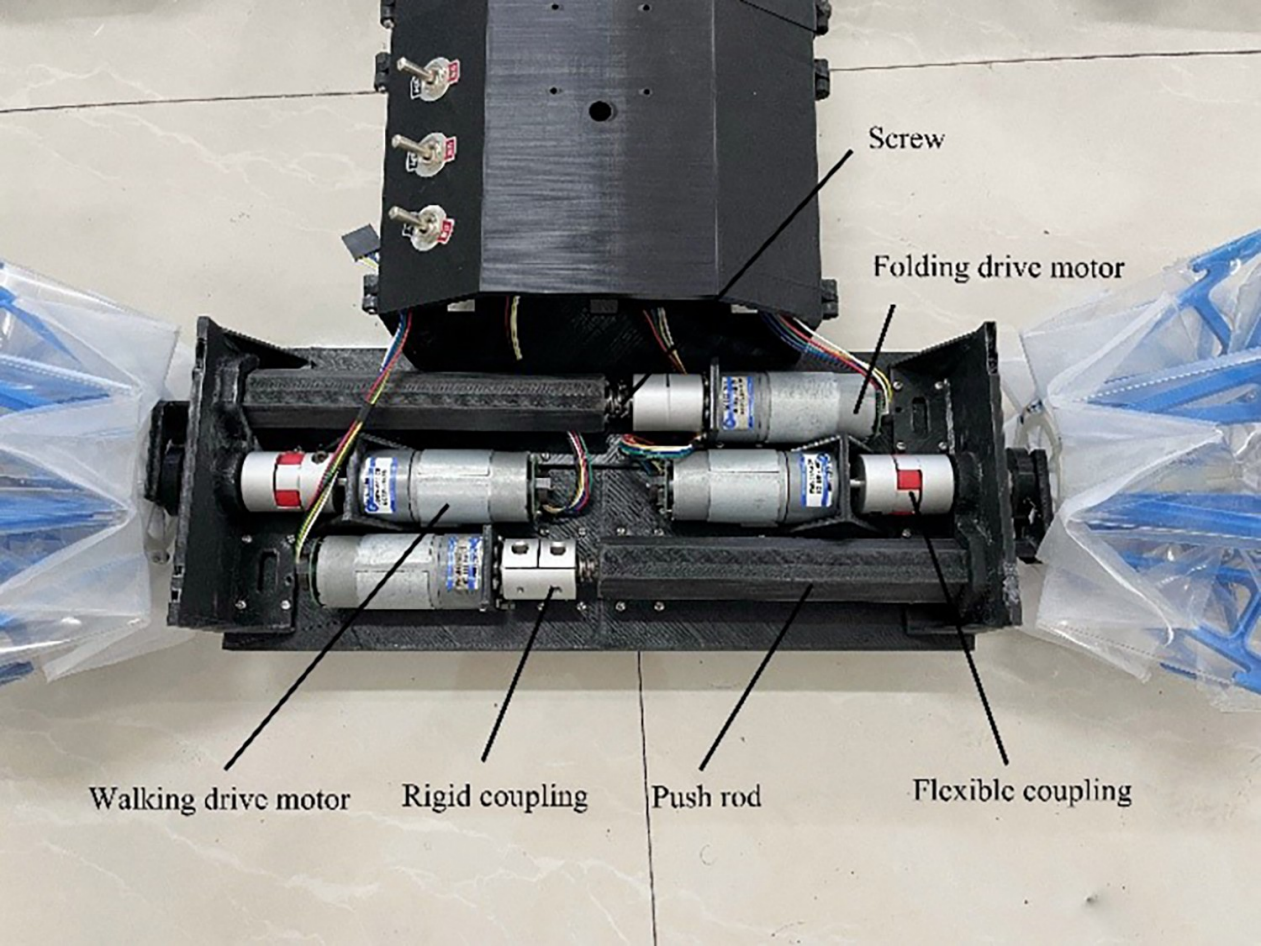
Structure of the whole robot.

**Fig 12:**
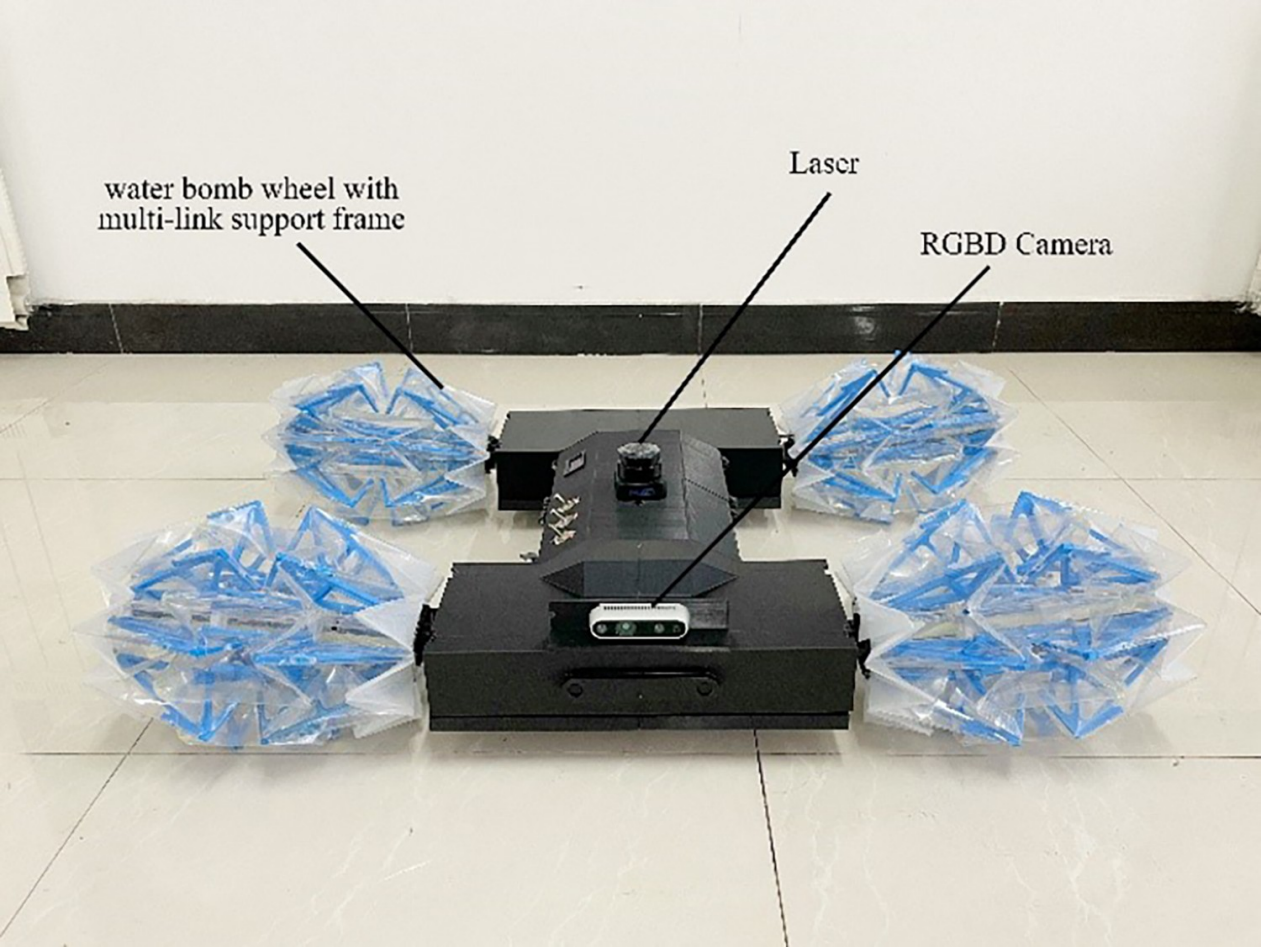
Robot drive system structure.

The robot has four variable diameter wheels, each of which is composed of a multi-link support structure and a water-bomb shell. During the entire folding process, the multi-link structure can be completely fitted with the water-bomb shell, and the wheel can complete the folding action by changing the distance between the two end faces. The robot has two sets of drive systems, namely the folding drive system and the walking drive system. The walking drive system connects the variable diameter wheel spindle to the motor through a coupling, and drives the vehicle to move by rotating the wheel. The folding drive system is driven by a motor to rotate the screw, which drives the push rod to extend and retract. The end of the push rod is connected to the inner end face of the variable diameter wheel through a fork, and the variable diameter wheel is folded by the motion of the push rod. The overall structure of the robot is shown in Fig 11, and the structure of the drive system is shown in Fig 12.

The maximum cross-section of the robot’s wheel is an octagon with a diagonal distance of D. Without considering the slipping phenomenon, it is the same as walking with a circular wheel. The walking process of a single wheel can be seen as a regular octagon rolling on the ground. To simplify the kinematic model, the octagonal wheel with a diagonal distance of D is equivalent to a circular wheel with the same circumference. The diameter of the equivalent circular wheel, d, is:

$$d=\frac{8D\sin(\pi /8)}{\pi }$$

**Fig 13:**
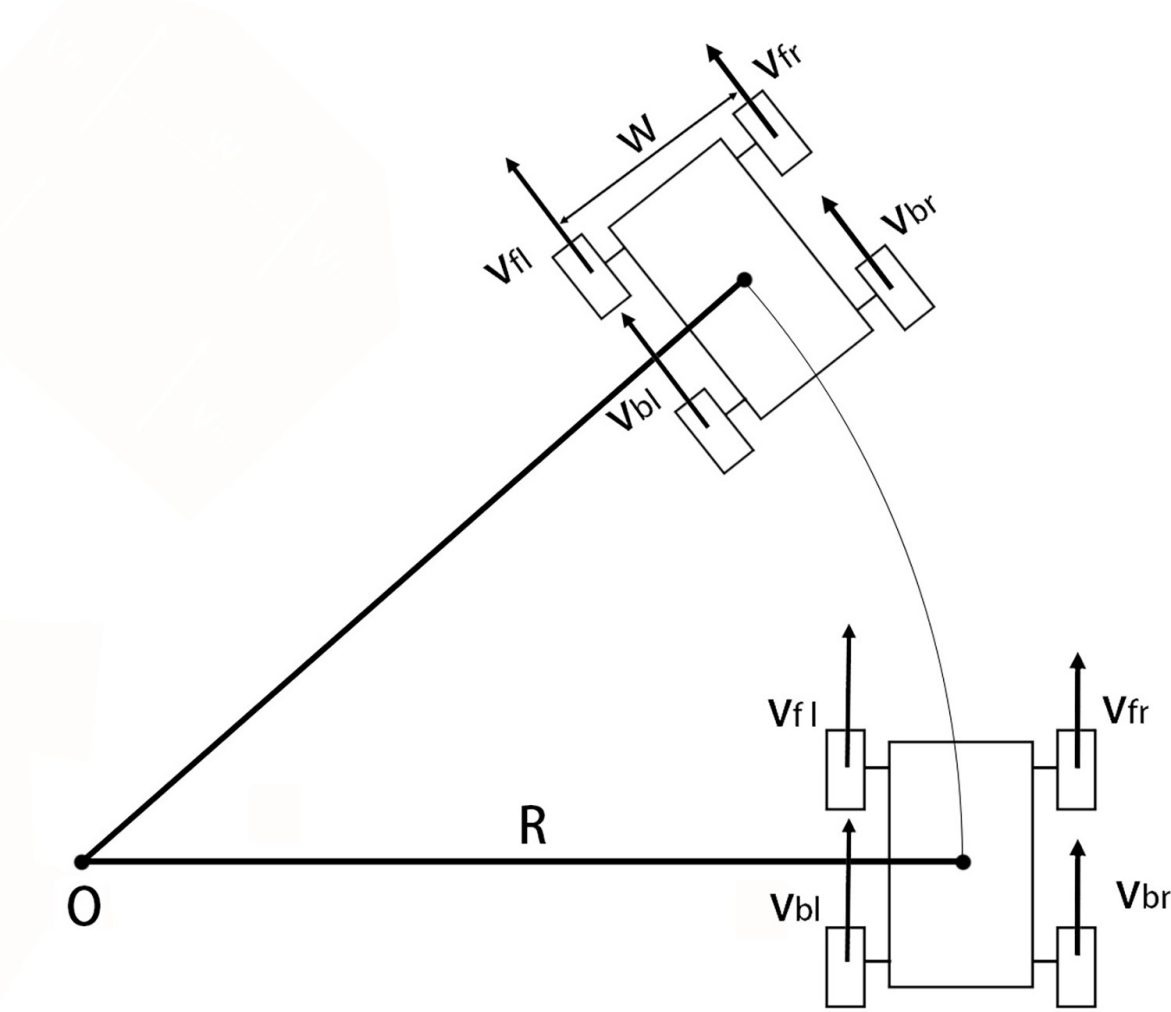
Robot kinematics solution of equivalent wheel diameter.

The robot performs kinematic analysis with an equivalent wheel diameter of d (as shown in Fig 13). During the motion, the speed of the left two wheels is *v*_*fl*_ = *v*_*fr*_ = *v*_*l*_, and the angular speed is *ω*_*l*_. The speed of the right two wheels is *v*_*fr*_ = *v*_*br*_ = *v*_*r*_, and the angular speed is *ω*_*r*_. The distance between the left and right wheels is *w*, the rotation radius is *R*, and the angular velocity of the robot center is *ω*. The following equations hold:

$$\left[\begin{array}{l} \omega_{l}\\ \omega_{r} \end{array}]=2[\begin{array}{cc} \frac{1}{d} & \frac{1}{2d}\\ \frac{1}{d} & -\frac{1}{2d} \end{array}][\begin{array}{c} v\\ w\omega \end{array}\right]$$

**Fig 14:**
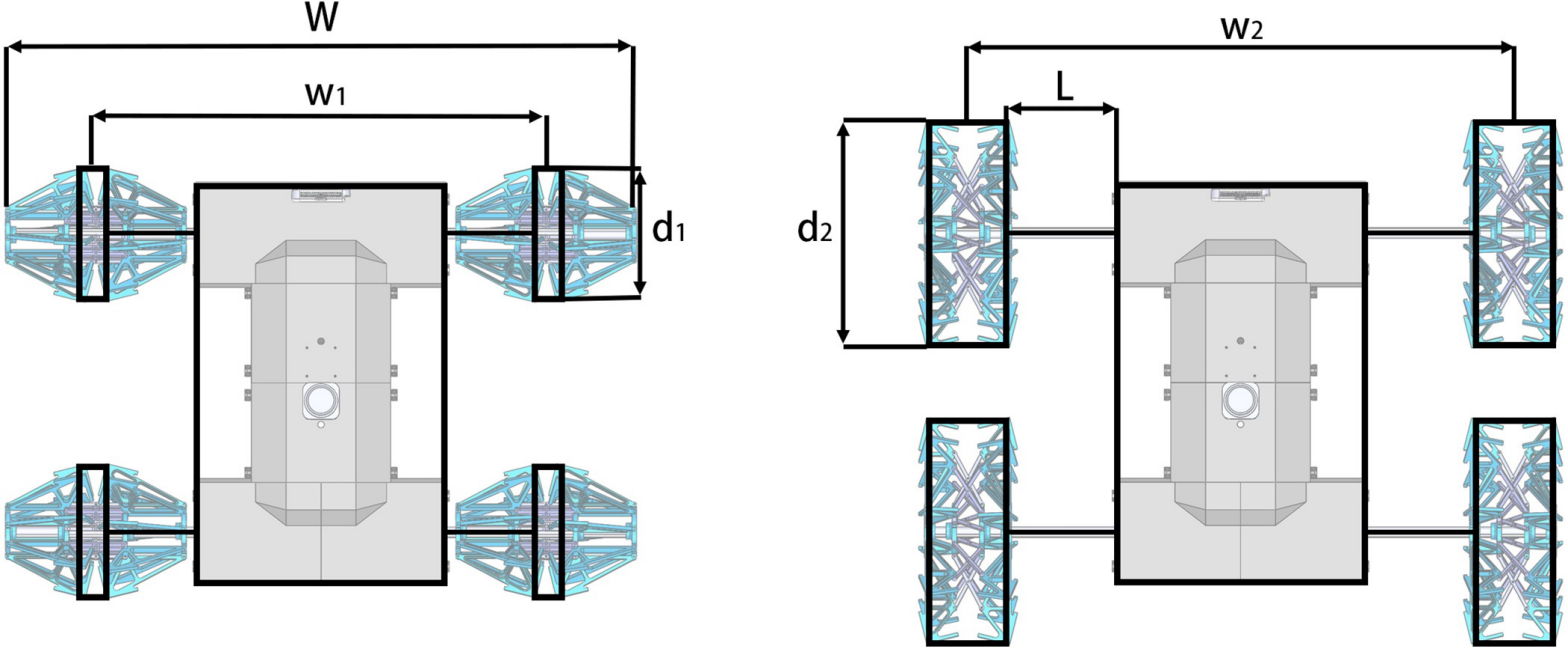
Schematic diagram of robot folding size parameters.

The wheeled robot has the characteristic of variable wheel diameter, and the wheel distance *w* also changes at the same time when the wheel diameter changes (as shown in Fig 14). The distance between the outer end surfaces of the left and right wheels of the robot is *W*. *L* is the extension length of the push rod. Using *L* as a state parameter and inputting it into the kinematic solution, the parameterized kinematic solution model of the robot can be obtained:

$$[\begin{array}{c} \omega_{l}\\ \omega_{r} \end{array}]=\frac{[\begin{array}{cc} 2 & 2\\ 2 & -1 \end{array}][(W-\sqrt{17}s-\frac{L}{2})\omega ]\frac{2}{68\sqrt{2}\pi s^{2}}}{\sin[\cos^{-1}(1-\frac{0.5L}{\sqrt{17}s})+2\tan^{-1}(1/4)]}$$

### Robot IMU-fused parametric odometer

The odometer refers to the position and direction of the robot relative to the world coordinate system, which is accumulated and calculated by the incremental Hall encoder driven by the walking driving motor from the moment the robot is powered on, with the assumption that the robot is located at the origin of the world coordinate system. Under the above assumption, the left front and rear wheels of the robot move synchronously, and so do the right front and rear wheels. Let the control cycle of the robot be *Δt* (*Δt = 10ms* in this paper). At time *k-1*, the yaw angle of the robot is *θ*_*k-1*_, and the robot is located at (*x*_*k-1*_, *y*_*k-1*_). At time *k*, the yaw angle of the robot is *θ*_*k*_, and the robot is located at (*x*_*k*_, *y*_*k*_). Each pulse of the encoder corresponds to the rolling distance of the equivalent circular wheel with a diameter of *d*, which is *Δl*_*per*_. The total number of pulses for one revolution of the wheel is *n*_*sum*_. The encoder values for the left and right sides at time *k* are *n*_*lk*_ and *n*_*rk*_, respectively, and at time *k-1*, they are *n*_*lk-1*_ and *n*_*rk-1*_. The incremental encoder values for the left and right sides are *Δn*_*l*_ and *Δn*_*r*_, respectively. The odometer calculation for the encoder at time *k* is given by:

$$\left[\begin{array}{c} x_{k}\\ y_{k}\\ \theta_{k} \end{array}]=[\begin{array}{c} x_{k-1}+\frac{\Delta n_{l}+\Delta n_{r}}{2}\Delta l_{p}\cos(\theta_{k-1})\\ y_{k-1}+\frac{\Delta n_{l}+\Delta n_{r}}{2}\Delta l_{p}\sin(\theta_{k-1})\\ \theta_{k-1}+\frac{(\Delta n_{r}-\Delta n_{l})\Delta l_{p}}{w} \end{array}\right]$$

$$\{\begin{array}{l} \Delta l_{p}=\frac{\pi d}{n_{\mathrm{sum}}}\\ d=\frac{8D\sin22.5}{\pi }\\ \Delta n_{l}=n_{\mathrm{lnow}}-n_{\mathrm{llast}}\\ \Delta n_{r}=n_{\mathrm{rnow}}-n_{\mathrm{rlast}} \end{array}$$

Because the robot may experience slipping during acceleration, deceleration, and turning, in order to further improve the accuracy of the odometer, the parameterized odometer information needs to be fused with IMU information. The IMU sensor uses a low-cost MPU6050, which integrates 3-axis MEMS gyroscope and 3-axis MEMS accelerometer. [*a*_*xk*_, *a*_*yk*_, *a*_*zk*_]^T^ and [*α*_*xk*_, *α*_*yk*_, *α*_*zk*_]^T^ are the three-axis acceleration and three-axis angular acceleration at time *k*, respectively. *x*_*ik*_, *y*_*ik*_, and *θ*_*ik*_ are the position and yaw angle observed by the IMU at time k. *v*_*xk*_, *v*_*yk*_, and *ω*_*k*_ are the x-direction velocity component, y-direction velocity component, and angular velocity of yaw angle at time *k*.


$$\left[\begin{array}{c} v_{xk}\\ v_{yk}\\ \omega_{k} \end{array}]=[\begin{array}{c} v_{xk-1}+\Delta t\cdot a_{xk}\\ v_{yk-1}+\Delta t\cdot a_{yk}\\ \omega_{k-1}+\Delta t\cdot \alpha_{zk} \end{array}\right]$$

$$\left[\begin{array}{c} x_{ik}\\ y_{ik}\\ \theta_{ik} \end{array}]=[\begin{array}{l} x_{ik-1}+\Delta t\cdot v_{xk}\\ y_{ik-1}+\Delta t\cdot v_{yk}\\ \theta_{ik-1}+\Delta t\cdot \omega_{k} \end{array}\right]$$

This is a technical description of a system for fusing data from an IMU (Inertial Measurement Unit) and an odometry sensor to improve the accuracy of the odometry estimate. At time *k*, the system defines the state vector *X*, observation vector *Z*_*k*_, state transition matrix *F*, process noise covariance matrix *Q*, measurement matrix *H*, and measurement noise covariance matrix *R*. The covariance values *r*_*1*_ to *r*_*3*_ represent the odometry covariance, while *r*_*4*_ to *r*_*6*_ represent the IMU covariance. The system uses these matrices and vectors to estimate the robot’s position and orientation more accurately than would be possible using odometry data alone.


$$X={\left[x,y,\theta \right]}^{T}$$

$$Z_{k}={\left[x_{k},y_{k},\theta_{k},x_{ik},y_{ik},\theta_{ik}\right]}^{T}$$

$$F=[\begin{array}{l} 1,0,0\\ 0,1,0\\ 0,0,1 \end{array}],Q=[\begin{array}{l} q_{1},0,0\\ 0,q_{2},0\\ 0,0,q_{3} \end{array}]$$

$$H=[\begin{array}{l} 1,0,0,0,0\\ 0,1,0,0,0\\ 0,0,1,0,0\\ 0,0,0,1,0\\ 0,0,0,0,1 \end{array}],R=[\begin{array}{l} r_{1},0,0,0,0,0\\ 0,r_{2},0,0,0,0\\ 0,0,r_{3},0,0,0\\ 0,0,0,r_{4},0,0\\ 0,0,0,0,r_{5},0\\ 0,0,0,0,0,r_{6} \end{array}]$$

The fusion process consists of a prediction step and an update step. The predicted state vector is Xp and the predicted covariance matrix is Pp:

$$\begin{array}{c} X_{p}=FX\\ P_{p}=FPF^{T}+Q \end{array}$$

The fused position and heading angle are stored in X, and the update steps are as follows:

$$\begin{array}{c} K=P_{p}H^{T}{\left(HP_{P}H^{T}+R\right)}^{-1}\\ P=(E_{3\times 3}-KH)P_{P}\\ X=X_{p}+K(Z-HP_{p}) \end{array}$$

## Robot SLAM mapping and autonomous navigation experiment

### Virtual environment experiment

**Fig 15:**
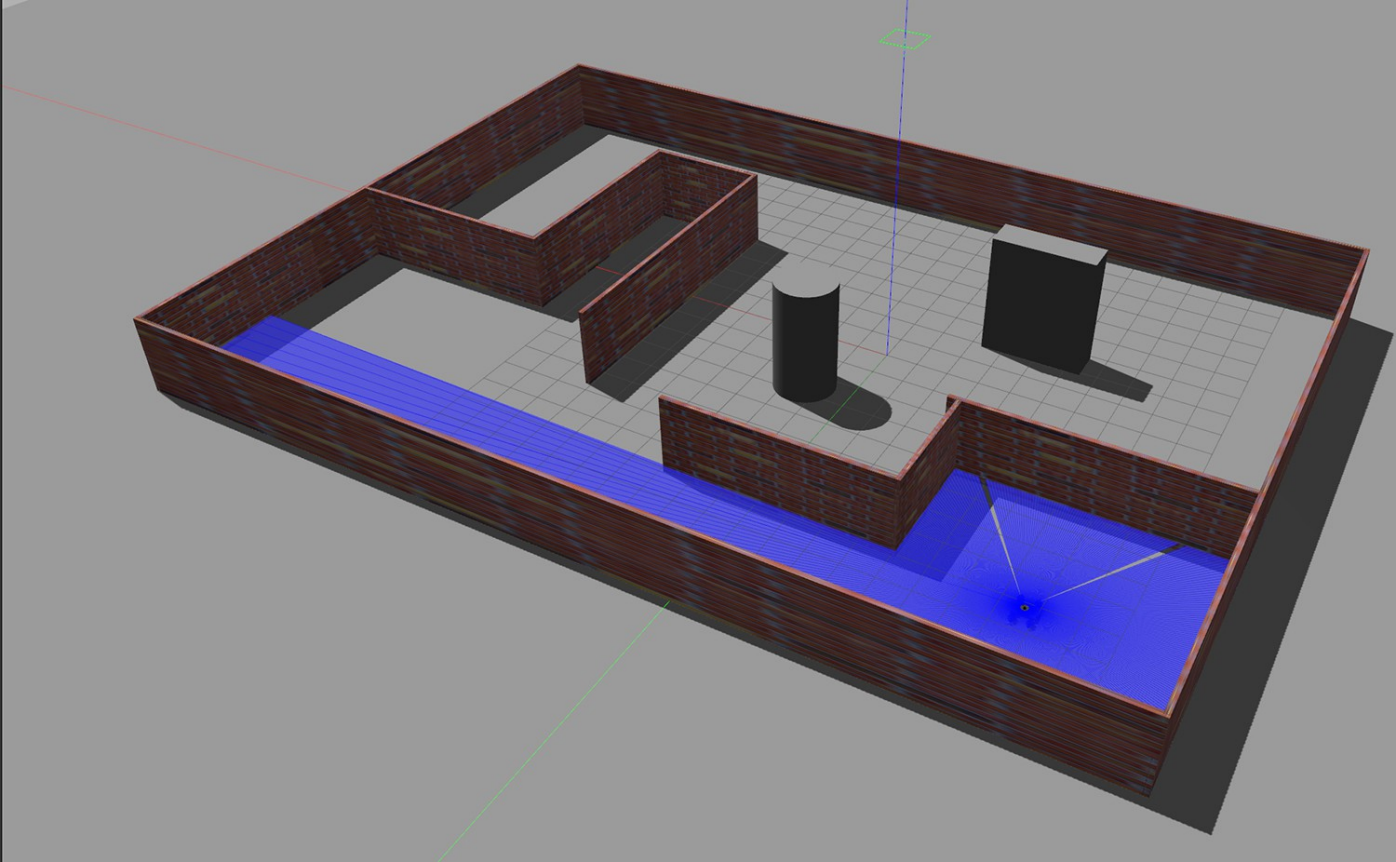
Gazebo virtual simulation environment.

In the Gazebo environment, a virtual space measuring 30m x 40m was constructed using the building editor, featuring several fixed obstacles, as shown in Fig 15. The three-dimensional model of the robot was configured with relevant link properties and component masses, and the robot model was exported as a URDF description file with inertia matrices. Using macro commands, the URDF file was further converted into an Xacro file representation. The Xacro-based robot model was then integrated into the virtual indoor environment. Realistic friction parameters were set in accordance with the actual environment to better simulate the robot’s motion in a real-world setting.

**Fig 16:**
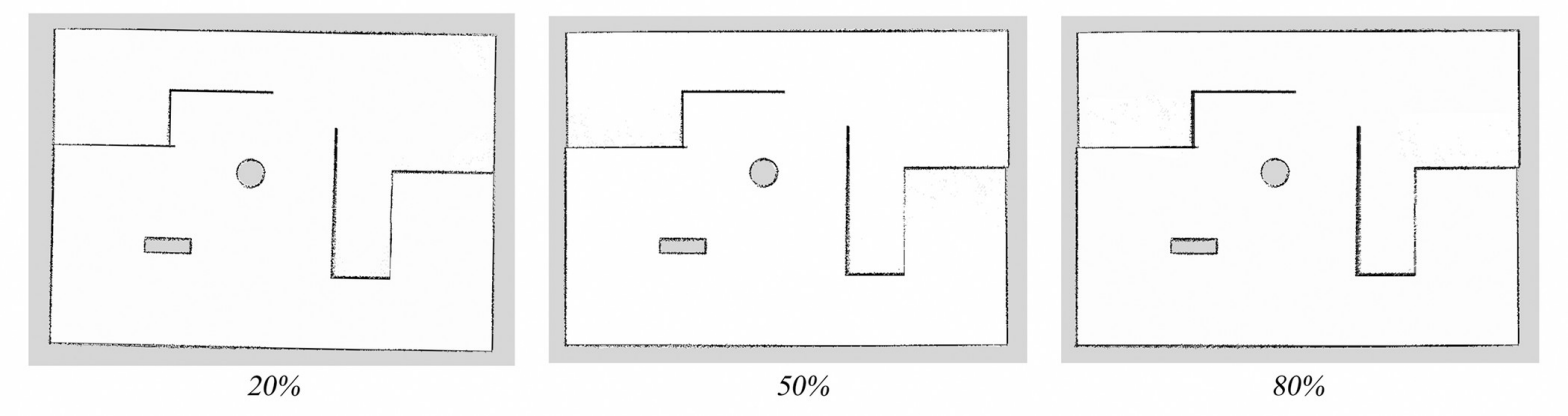
SLAM mapping results in virtual environment.

**Fig 17:**
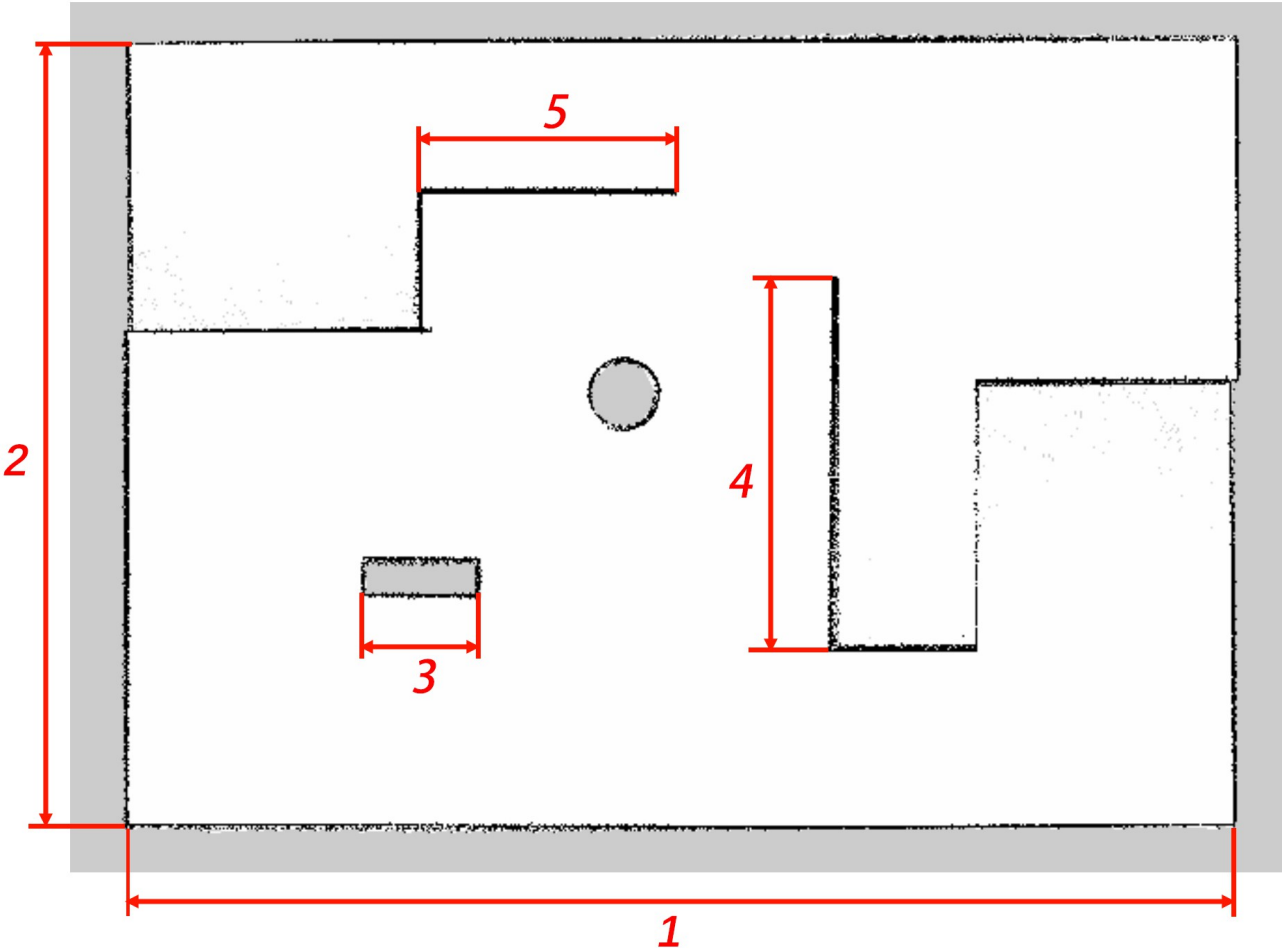
Virtual environment landmark location.

The mapping algorithm, incorporating parametric kinematic solutions and IMU-fused parametric odometry, was employed to construct the maps. Environmental maps of the virtual experimental field were generated under different wheel diameter state (20%, 50%, and 80% wheel expansion), with a uniform grid size of 1 cm for all three maps. The mapping results are presented in Fig 16. As illustrated in Fig 17, five marked locations, denoted as Landmark 1 to Landmark 5, were quantitatively assessed. The map size were based on the grid size in the map, and the absolute error values were summarized, as detailed in Table 1. Mapping errors under all three wheel diameter state were found to be below 3%. This demonstrates that the mapping algorithm, utilizing parametric kinematic solutions and IMU-fused parametric odometry, produces maps characterized by high mapping accuracy.

**Table 1:** Virtual environment map quantitative evaluation data.

Landmark	Virtual environment size	Map size			Absolute value of error		
		20%	50%	80%	20%	50%	80%
1	4000cm	4043cm	4070cm	4095cm	43cm	70cm	95cm
2	3000cm	3035cm	2959cm	2913cm	35cm	41cm	87cm
3	425cm	419cm	416cm	414cm	6cm	9cm	11cm
4	1345cm	1313cm	1318cm	1377cm	25cm	27cm	32cm
5	925cm	910cm	905cm	948cm	15cm	21cm	23cm

**Fig 18:**
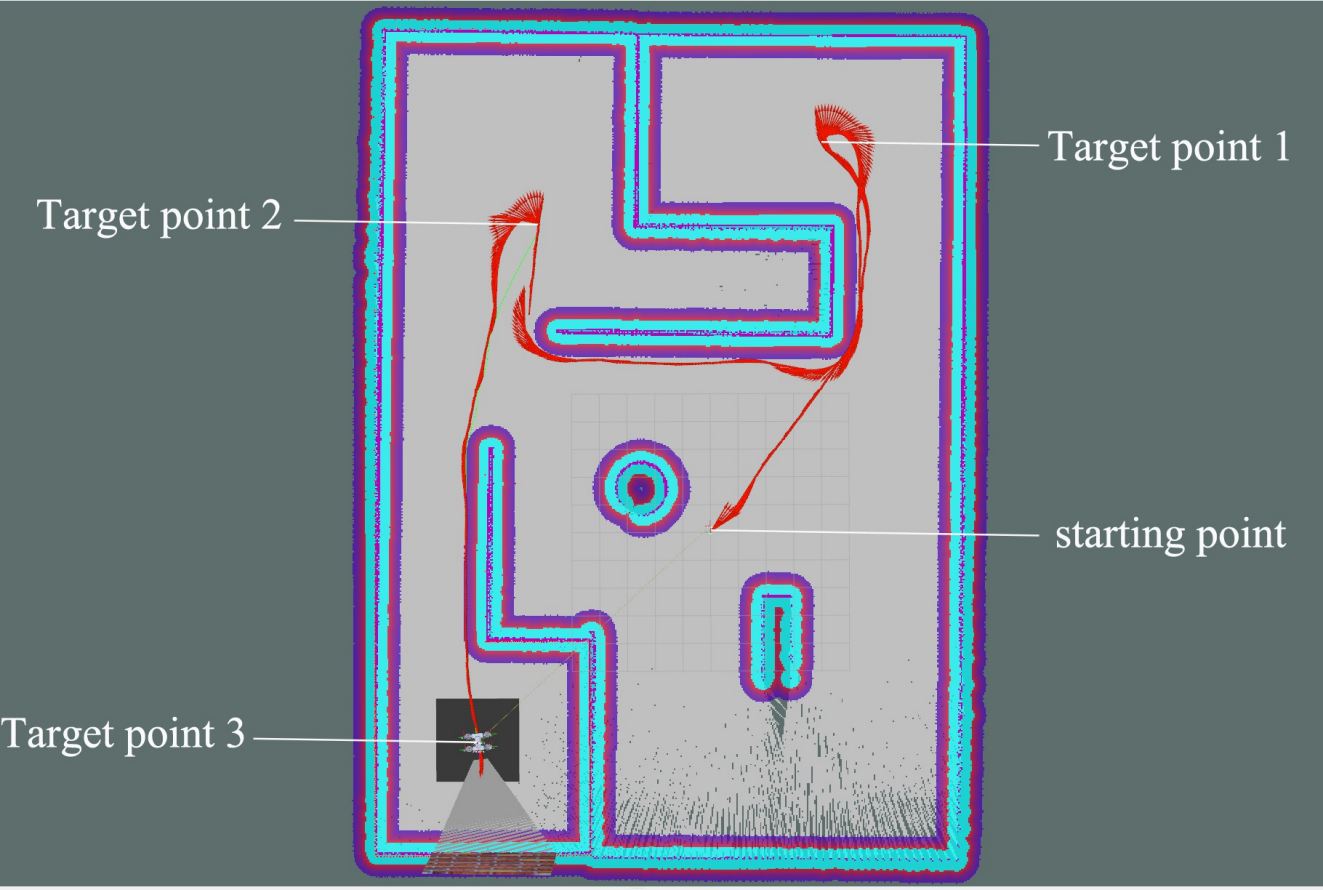
Autonomous navigation experiment in virtual environment.

During the path planning experiment, the robot was instructed to reach three specified target locations on the global map in sequence, and its movement path was displayed in red arrows in Rviz using odometry. As shown in Fig 18, it can be seen that the robot always took the shortest path to the target and effectively avoided fixed obstacles. The result illustrated the parameterized kinematic solutions and IMU-fused parameterized odometry are effective and accurate in the robot autonomous navigation under virtual environment.

### Laboratory environment experiment

**Fig 19:**
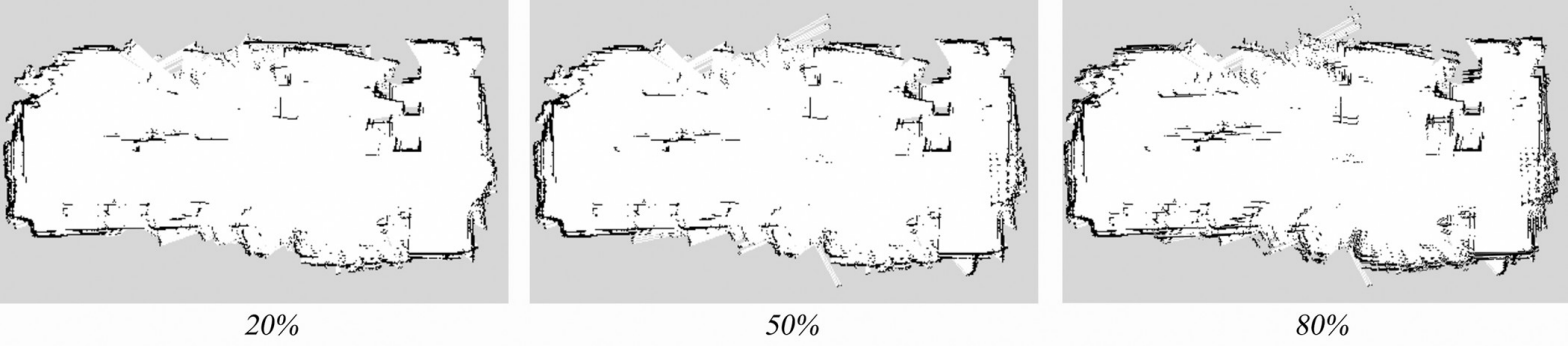
Map construction effect without parameterized kinematic solutions and IMU-fused parameterized odometry.

**Fig 20:**
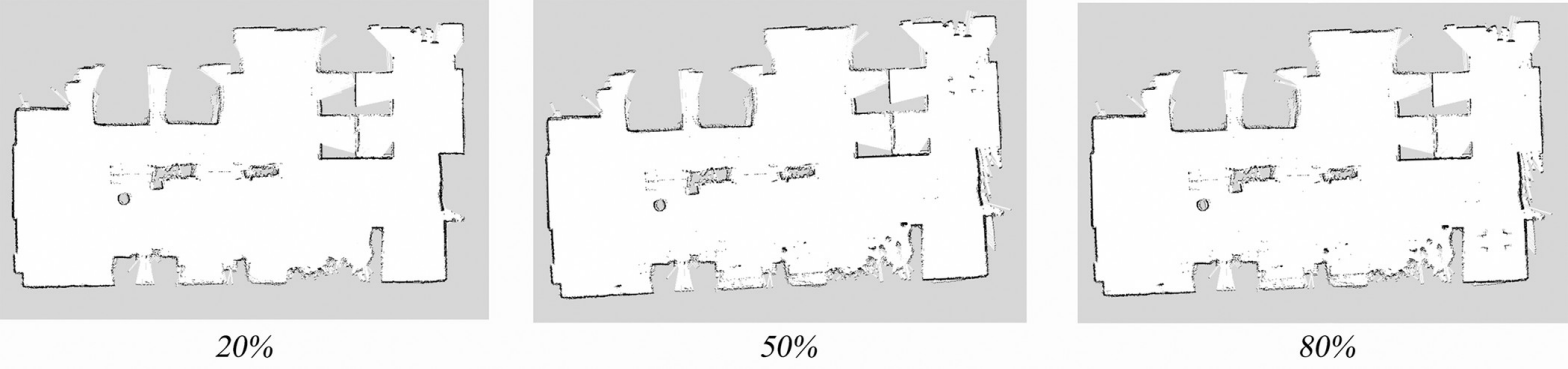
Map construction effect with parameterized kinematic solutions and IMU-fused parameterized odometry.

Laboratory environment maps were constructed under different wheel diameter state (wheels expansion to 20%, 50%, and 80%, respectively). Maps were created using the mapping algorithm that incorporates parameterized kinematic solutions and IMU-fused parameterized odometry. Traditional mapping algorithms were also used to create maps. Comparing the two approaches, it can be observed that the maps constructed by the traditional mapping algorithm exhibit significant misalignment, as shown in Fig 19. In contrast, the maps created by the mapping algorithm that incorporates parameterized kinematic solutions and IMU-fused parameterized odometry demonstrate excellent mapping accuracy, as depicted in Fig 20.

**Fig 21:**
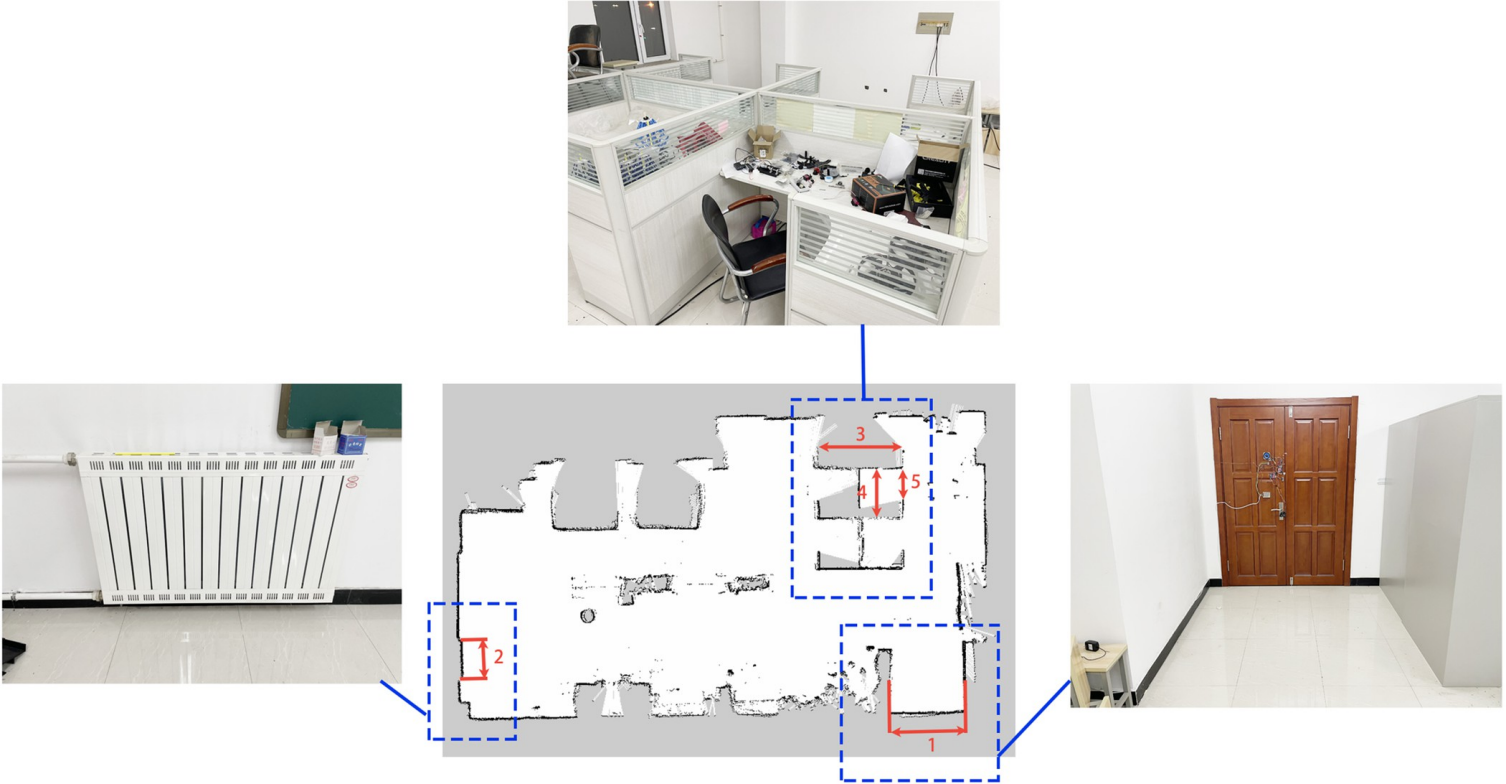
Laboratory environmental landmark location.

Quantitatively evaluate the grid maps which constructed with the mapping algorithm that incorporates parameterized kinematic solutions and IMU-fused parameterized odometry under three different wheel diameters state. The grid size for all three maps was set to 1mm. Five representative landmark obstacles in the maps were selected, as shown in Fig 21. The numbered markers in the figure correspond to landmarks labeled as 1 to 5. The actual sizes value were the average value of 3 measurements of landmark obstacles by laser rangefinder, the map size were the average value of 3 maps under each different wheel diameters state based on the grid size. The actual size, map size, and absolute error of landmarks 1 to 5 were summarized, and the data are presented in Table 2. From the data, it can be calculated that the map sizes of several landmark obstacles have errors smaller than 5% compared to the actual size. This indicates that the introduced fusion of IMU information with parametric odometry and parametric kinematic solution leads to higher accuracy in the constructed maps.

**Table 2:** Laboratory environment map quantitative evaluation data.

Landmark	Actual size	Map size			Absolute value of error		
		20%	50%	80%	20%	50%	80%
1	2010mm	2030mm	1967mm	1940mm	20mm	43mm	70mm
2	1155mm	1145mm	1170mm	1110mm	10mm	25mm	45mm
3	2540mm	2513mm	2586mm	2480mm	27mm	46mm	60mm
4	1500mm	1488mm	1522mm	1555mm	12mm	22mm	55mm
5	940mm	955mm	971mm	980mm	15mm	31mm	40mm

**Fig 22:**
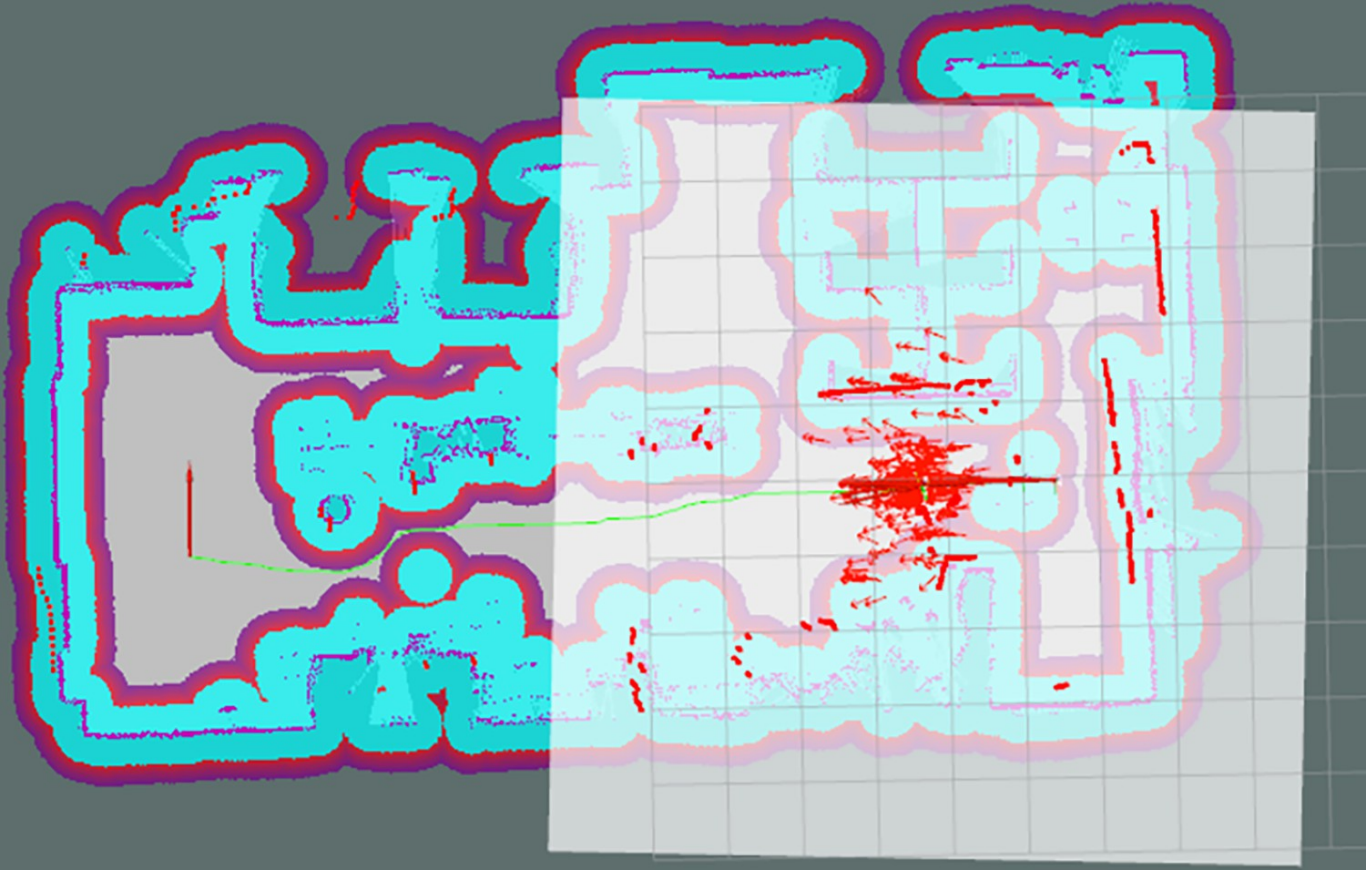
Autonomous navigation experiment in laboratory environment.

Experiments were conducted on the autonomous navigation of the robot, in which a target point was specified on the map for the robot to reach autonomously. The green line in Fig 22 shows the global path of the robot. The autonomous navigation of the physical robot can meets expectations, illustrated the parameterized kinematic solutions and IMU-fused parameterized odometry are effective and accurate in the robot autonomous navigation under actual environment.

## Summary and outlook

This paper presents a recursive parameterized modeling method for water-bomb wheels. Compared to traditional methods, this recursive parameterized modeling approach doesn’t require the use of global optimization solvers to solve large sets of equations. It only involves recursively solving several sets of equations, each composed of three equations. Therefore, it reduces the complexity of the solving process and enhances precision by eliminating the need for global optimization solvers.

Building upon this model, a deformable wheel was designed by combining a multi-link support structure with the water-bomb wheel. The effectiveness of the multi-link structure’s support was verified through Abaqus simulations and experiments. For a robot equipped with this deformable wheel, a mapping algorithm that incorporates parameterized kinematic solutions and IMU-fused parameterized odometry was developed, which is used for Simultaneous Localization and Mapping (SLAM) and autonomous navigation. Experiments were conducted in both a virtual Gazebo environment and a real laboratory environment with the wheels diameter at 20%, 50%, and 80% states, and the performance of the SLAM system was assessed. The experimental results demonstrated that the SLAM mapping errors were all below 5% in both virtual and laboratory environments, validating the algorithm’s effectiveness.

Additionally, a comparison was made in the laboratory environment between the mapping algorithm that incorporates parameterized kinematic solutions and IMU fusion and a traditional algorithm. The results showed that the algorithm used in this paper significantly improves mapping accuracy. Future research will focus on 3D SLAM and robot obstacle avoidance strategies to achieve autonomous obstacle avoidance functionality.

## Supporting information

S1 File Water-bomb modle. (GH)

S2 File Water-bomb shell. (DWG)

S1 Video Robot video. (MP4)
